# Cognitive and Affective Processing of Risk Information: A Survey Experiment on Risk-Based Decision-Making Related to Crime and Public Safety

**DOI:** 10.3389/fpsyg.2020.02222

**Published:** 2020-09-04

**Authors:** Colleen M. Berryessa, Joel M. Caplan

**Affiliations:** School of Criminal Justice, Rutgers University, Newark, NJ, United States

**Keywords:** risk, information processing, dual-process, decision-making, cognitive, affective, policing

## Abstract

The current study, using a multi-factorial survey experiment with a sample of the general public (*N* = 800), investigates if and how types of risk information on crime and public safety, such as maps, graphs, or tables, commonly used and communicated by law enforcement elicit dual-process (affective and cognitive) risk information processing in risk-based decision-making, and if such processing or decision-making differs depending on the risk level, context, or the type or format of risk information communicated. Participants responded to a vignette in which they were asked to choose a ride-share pick-up point within a certain geographic area with varying risk levels of being involved in a pedestrian-automobile crash. Results showed that risk information related to crime and public safety elicits dual-process risk information processing, and that both affective and cognitive processing significantly predicted risk-based decision-making, regardless of the risk level or type of risk information examined. Interestingly, risk information was used to create an almost “black and white” distinction for participants, in which their lowest-risk choice was treated as their comparison point, relative to all other higher levels of risk, in risk processing and decision-making. Further, the risk level or type of risk information examined did affect the nature and level of affective and cognitive processing elicited, suggesting that different types or characteristics of risk information can change modes of processing and their effects on risk-based decisions. Our findings provide first-of-its-kind data that show members of the general public, as consumers of risk information in relation to crime and public safety, process and make decisions surrounding such information using the dual-process approach. Implications for communicating risk information in relation to crime and public safety to both the general public and police, as well as how to extend the current inquiry to future areas of research on police, are discussed.

## Introduction

Policing has evolved to play a central and pivotal role in managing and communicating information related to crime and public safety (Cope, 2004). The use of data-driven and evidence-based strategies, which have become a cornerstone for efficient and effective law enforcement operations, is not new (Coldren et al., 2013; Randol, 2014). In the 19th century, Sir Robert Peel called on law enforcement to keep and collect data, both to inform the public of crime and public safety conditions and to target the distribution of resources (Gaines et al., 1999). Since Sir Robert’s time, demands on technology and information have changed; however, the core mission remains: information on crime and public safety should be collected, organized, analyzed, and distributed to facilitate informed risk-based decision-making by both the police and the public (O’Shea and Nicholls, 2003). In the spirit of Sir Robert, the current study looks to investigate if and how types of risk information on crime and public safety, such as maps, graphs, or tables, influence risk-based decision-making by the public. Specifically, using a multi-factorial survey experiment with a sample of the general public (*N* = 800), we were interested in seeing if risk information elicits dual-process (affective and cognitive) risk information processing in such decision-making, and how processing and decisions may differ depending on the risk level, context, or the type or format of risk information communicated.

Modern data-driven police operations generally involve different types of information processing related to crime and public safety, including counts, trends, pattern and series identification, and different types of mapping (Reuland, 1997; Dağlar and Argun, 2016). In addition to informing the public about crime and public safety trends and conditions, information processing is also key to numerous contemporary law enforcement strategies, including problem-oriented policing (Scott, 2000; Carson and Wellman, 2018), hotspot policing (Braga et al., 2019), risk-based policing (Kennedy et al., 2018) and CompStat (Weisburd et al., 2003; Miller, 2008; Pasha and Kroll, 2016). Thus, maps, graphs, charts, and other data are crucial tools for both future planning and real-time decision-making related to crime and public safety (Bonkiewicz, 2015; Davis et al., 2015; Caplan and Kennedy, 2016; Wheeler, 2016).

The mechanics and analytic outputs related to data-driven police operations chiefly rely on the process of risk recognition (Goldsmith et al., 1999; Mesch, 2000; O’Shea and Nicholls, 2003). In our “risk society,” law enforcement have become the central store of risk-based knowledge for fellow law enforcement and the public (Campbell, 2004; Ericson, 2007). Indeed, one of the primary focuses of police operations is *risk communication*, which emphasizes the understanding and assessment of risk, as it relates to both crime and public safety, through data and information (Ericson and Haggerty, 1997; Gutteling and Wiegman, 2013). This involves processing and synthesizing different types and formats of risk information, showing what happened and where, in order to influence the choices made in response to this risk information (Ericson and Haggerty, 1997; Gutteling and Wiegman, 2013).

For example, when a current crime trend is identified as being high in one area relative to other locations, a police department may adopt specific strategies in an attempt to reduce and deter future criminal activity in that specific location (Chalfin and McCrary, 2017; Kelly, 2019; Baughman and Caplan, 2020). Indeed, falls in crime counts can be used as a performance tool to see if a particular strategy has effectively decreased risk in a certain area (Braga and Weisburd, 2010). For the public, police-provided risk information illustrating trends on crime and public safety conditions may affect their perceptions and corresponding decisions on what areas of a city or features of a landscape to frequent or avoid (Caplan, 2011; Roberts, 2018).

As individuals have their own perspectives on risk communication, risk information in relation to crime and public safety can often take on different forms (Spiegelhalter, 2017; Lundgren and McMakin, 2018). Crime and public safety data are often presented in tables, graphs, and through numerical counts, and such modes are frequently presented internally by law enforcement agencies and externally for crime reporting purposes and public awareness (Santos, 2014; Santos and Taylor, 2014; Wheeler, 2016). Tables, graphs, and numerical counts help the consumers of such information to monitor temporal crime trends and visualize data during risk recognition and related decision-making (Guilfoyle, 2016; Wheeler, 2016). Further, numerical risk information is often provided for varying geographic or jurisdictional distances (i.e., street-level, neighborhood-level, city-level, county-level, or state-level) in order to contextualize and compare types of risk from one place to another (Pridemore, 2005; Deane et al., 2008; McDowall and Loftin, 2009).

Mapping has also developed into a major tool for crime analysis (Block et al., 1995; Caplan and Moreto, 2012; Roth et al., 2013; Roth et al., 2015). Maps help provide visual risk-detail on a geographic location and can include details, such as street names and locations of significant events, related to risk-based decision-making; maps can be presented as more realistic satellite images, which may include terrain or images of landmarks, or as street maps which show a basic street outline of a general area (Harries, 1995; Harries and LeBeau, 2007). Sometimes maps are presented with other types of risk information. For example, a map of a geographic area that is high in assault offenses could be presented alongside a table with related crime counts (Harries, 1995). Other maps, rather than including numerical counts, may include line weights, symbols, colors, and shading to provide risk information. Indeed, for example, hot spot maps use plotting density of different colors and shades to indicate the number of crimes or incidences in particular areas (Eck et al., 2000; Weisburd et al., 2012). Risk terrain maps (Caplan et al., 2011a; Kennedy et al., 2011; Caplan and Kennedy, 2016) identify places where the interaction effects of environmental generators (i.e., areas that attract a large number of people, such as parks or malls) and attractors (i.e., areas with known opportunities for criminal behavior that attract individuals with previous criminal histories and a high motivation to offend) of illegal behavior increase the risk of crime emergence or persistence. Different colors, line weights, or shades help consumers of such maps, whether they be police or the public, to visually “sort out” different levels of risk during information processing and decision-making (Harries, 1995; Chainey and Tompson, 2008; Kennedy et al., 2011; Caplan et al., 2020).

Yet, the impact of risk recognition and information processing on decision-making may not be “one size fits all” for consumers of crime and public safety data, including both law enforcement and the general public (Carter et al., 2017). Indeed, risk information processing of public safety and crime data likely differ depending on the type or format of risk information, such as maps, graphs, or tables, the level of risk being weighed, or the potential consequences of risk-based decisions (Harries, 1995; Lundgren and McMakin, 2018). Indeed, there is already significant evidence that distinctive modes of risk information processing may be elicited in response to different characteristics and contexts of risky situations (Van Gelder’s et al., 2009; Caplan et al., 2011b; Piza et al., 2017).

Historically, risk recognition and decision-making in psychology was thought to be a set of mental calculations that only incorporated the probability of outcomes of a decision together with an evaluation of these outcomes (Vlek and Stallen, 1980; Hendrick et al., 1989; Visschers et al., 2012). However, more recent literature has concluded that risk recognition and decision-making is actually a combination of two different process modes: rapid, automatic, effortless, and affective information processing (System 1), and slower, deliberate, effortful, and cognitive information processing (System 2) (Kahneman, 2003; Slovic et al., 2005; Mukherjee, 2010). Risks are recognized and acted upon as both “risk-as-analysis” (i.e., System 2) and “risk-as-feelings” (i.e., System 1) (Slovic et al., 2005). This interplay between cognition and affect is called dual-process risk information processing (e.g., Kruglanski and Orehek, 2007; Evans, 2008; Wang et al., 2017).

Dual-process approaches in risk information processing have shown that both cognitive and affective processing interact and coexist in risk recognition and decision-making, but may respond to different characteristics of a situation (Lerner and Keltner, 2001; Hodgkinson and Clarke, 2007). Affective and cognitive modes of risk recognition and decision-making make independent contributions to risk-based decisions depending on the situation, context, and content of the decisions, specifically if they are high risk (Ferreira et al., 2006; Venkatraman et al., 2009). For example, cognitive processing may be more sensitive to analytic risk considerations, such as probabilities or counts, whereas affective processing is more likely to respond to visual, feeling-based considerations and may not be as responsive to probabilities (Slovic et al., 2005).

Indeed, as affective processing is unconscious, automatic, and effortless in nature, it involves recognizing and identifying risk based on visual cues and then matching cues to patterns from a person’s long-term memory (Allen and Thomas, 2011; Caplan, 2011; Thompson and Morsanyi, 2012). This process is often described as having a “gut feeling” and uses schemas to speed up risk-based decision-making (Kruglanski and Orehek, 2007; Evans, 2008). High-risk situations have also been found to elicit more affective reactions, as they may elicit decision-making influenced by fear (Loewenstein and Hsee, 2001). Indeed, “risk-as-feelings” can sometimes overpower cognitive evaluations of probability or numerically based risk recognition, which in turn can affect behavior in higher risk situations (Loewenstein and Hsee, 2001).

As studies have shown that both cognitive and affective modes of risk information processing influence risk recognition and decision-making depending on the level or contextual nature of the risk (Ferreira et al., 2006; Evans, 2008; Van Gelder’s et al., 2009), it is possible that consumers of common types and formats of risk information used for crime and public safety purposes, such as police command staff and the general public, may process and make decisions surrounding such information using the dual-process approach. Such an inquiry has not yet been undertaken.

Building upon this framework, the current study investigates if and how common types of risk information used to communicate crime and public safety conditions, such as maps, graphs, or tables, elicit dual-process risk information processing in risk-based decisions, and if such processing or decision-making differs depending on the risk level, context, or the type or format of risk information communicated. We use a multi-factorial vignette-based experimental survey design, which describes the risk of being involved in a pedestrian-automobile crash within a certain geographic area, with a research sample that is known to regularly consume this information: members of the general public.

This study largely acts as an exploratory inquiry with regards to how different risk levels, contexts, and information may affect risk-based decision-making in relation to crime and public safety. However, with regards to risk information processing, we did anticipate that both cognitive and affective processing would be significant predictors of risk-based decision-making, regardless of the risk level or type of risk information examined in the current study, but that affective processing (i.e., “risk-as-feelings”) would be more important in risk-based decision-making involving high levels of risk and more visual forms of risk information (i.e., risk information presented on maps). Indeed, when processing and synthesizing different types of basic risk information, we also expected cognitive processing to be more associated with risk-based decision-making involving probability or numerically based risk information, such as graphs, tables, or numerical counts.

## Materials and Methods

### Participants

The target population was U.S. adults. The sample for this study was drawn from the online crowd sourcing service Amazon’s Mechanical Turk (MTurk), which has become a popular and successful survey sample tool for social scientists in recent years (Barger et al., 2011; Rouse, 2015) and has been used in a variety of published vignette experiments across disciplines in recent years (e.g., Berryessa, 2017, 2018; Berryessa and Lively, 2019; Berryessa and Krenzer, 2020). Although it has its limitations in its use (see below in the study’s discussion), Amazon’s MTurk allows for the collection of inexpensive, convenient, fast, and diverse data (Buhrmester et al., 2011). Although exact numbers are unknown, MTurk has more than 500,000 registered “workers,” of which about 50% are members of the U.S. adult population, who complete different types of Human Intelligence Tasks (Stewart et al., 2015). In order to obtain the sample, a link to and short description of the study (built on Qualtrics) was posted to the MTurk online system, with a request for responses from 800 “workers” that are adult members of the U.S. population (age 18+, U.S. residing in the U.S.), and the survey was available to be taken by any MTurk worker who fit these sample criteria.

Respondents self-selected from that service to participate in the study, and were each randomly administered different versions of the vignette-stimuli (paid $1.00 USD each). After a sample of 800 individuals that fit these criteria participated in the study, no additional individuals were allowed to participate in the study and the survey was closed. An attention-check question was asked to ensure that respondents read and understood the vignette stimuli, as well as an honesty question that asked respondents if they had been truthful in their answers, and participants who did not correctly answer those questions were automatically excluded from participation in the middle of the study. Three participants were eliminated from analyses after failing the honesty or check questions. An IP blocker was used so respondents could take the survey once.

### Study Design and Procedure

This study is a 2 × 9 (+2) between-subjects, multi-factorial randomized online experiment utilizing vignette stimuli describing the risk of being involved in a pedestrian-automobile crash within a certain area, whereas the respondent would be the pedestrian. Our goal was not to only examine risk information processing and decision-making in relation to this specific scenario or situation, but to measure intuitions toward a realistic, recognizable risky situation in an attempt to illustrate how common forms of risk information in relation to crime and public safety are recognized, processed, and used in decision-making. Indeed, it is common to use vignettes involving personally relevant risky situations, such as drunk driving or automobile accidents, in risk-based experiments involving members of the general public (Van Gelder’s et al., 2009).

Our vignettes varied by (1) the type of map (satellite map or street map; both unlabeled); and (2) the type or format of risk information (numerical counts, table, bar graph, line graph, pie graph, hotspot map in color, hotspot map in grayscale, thematic map in color, and thematic map in grayscale) provided to each participant. There were also two control groups that varied by the type of map given to participants (satellite map or street map), but they did not provide any type of risk information to participants (control groups).

In total, there were 18 experimental vignette conditions and 2 control vignette conditions (an equal number of participants received each vignette condition). All stimuli not included in the main text are found in Supplementary Figures 1–10. Vignettes were pretested before data were collected. This study received approval from the Rutgers Institutional Review Board.

### Vignette Design and Measures

Respondents participated in the survey via a Qualtrics link posted to MTurk in March 2020, and the entire study took each participant, on average, 10.36 min. All participants were asked to provide informed consent. After providing consent, all participants were presented with the following information:

You are currently in Anytown, United States. You just called a ride share car service and you need to get picked up right away. Your current location is marked by the star on the map, and the circle on the map shows the pick-up radius. You have the option of meeting the driver at one of four different pick-up spots labeled on the map as North (N), South (S), East (E), and West (W). All four pick-up points (N, S, E, and W) are the same distance from your current location at the center of the map and are all a quarter of a mile walk. Every pick-up point is the same driving time to your ultimate destination. Motor vehicle crashes involving pedestrians are a major concern throughout Anytown, United States. All streets in Anytown, United States allow for both motor vehicle traffic and pedestrian foot traffic, and therefore, the potential for this problem exists everywhere in town. However, some places in Anytown, United States have more pedestrian crashes than other places.

**FIGURE 1:**
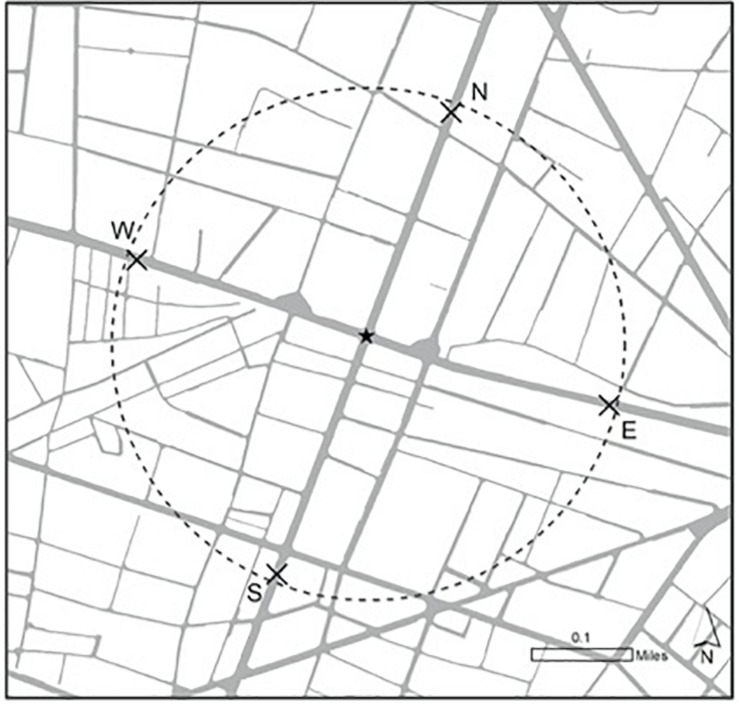
Street map of pick-up area.

**FIGURE 2:**
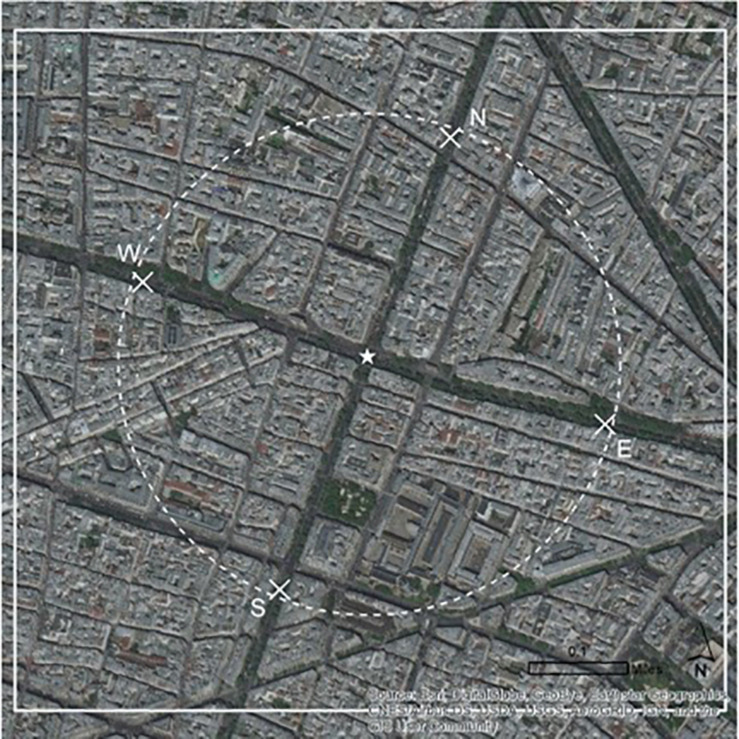
Satellite map of pick-up area.

Alongside that information, participants were randomly presented with either one of two maps of the pick-up area described above: (1) a street map showing the four-pick up spots (N, S, E, and W) and a star of the participants’ current location (see Figure 1), or (2) a satellite map showing the four-pick up spots (N, S, E, and W) and a star of the participants’ current location (see Figure 2). Both maps showed the same area, pick-up spots, and location, and solely differed on the format of the map (street map versus satellite map).

Next, alongside either the satellite map or street map they had received, all participants were told that there had been 216 motor vehicle crashes involving pedestrians in this quarter-mile pick-up area on the map within no particular time period (i.e., the temporal span of these incidents was ambiguous). Two control groups, although varying by the type of map, did not receive any further risk information about these 216 motor vehicle crashes and where they occurred. The control groups served to provide the researchers information on participants’ views of the map and pick-up area without their consideration of any risk information on the four pick-up spots.

Those participants that were not in the control groups were randomly presented with one of nine types of risk information on the 216 motor vehicle crashes involving pedestrians in the pick-up area and how many crashes had occurred near each of the four pick-up spots (North, South, East, and West). Although the risk information differed by type (see below), all risk information conveyed the same number of crashes on the way to each pick-up spot. These data were generated by the authors explicitly for the purpose of this study, and they are not real crash statistics associated with any particular place or time.

North: 67 pedestrian crashes

South: 43 pedestrian crashes

East: 85 pedestrian crashes

West: 21 pedestrian crashes

Thus, according to all experimental stimuli, the pick-up areas listed in order from highest to lowest risk of crashes are East, North, South, and West, respectively.

The nine different types of risk information—alongside either the satellite map or street map respondents had originally received—were all versions of common forms of risk information that are regularly communicated to both law enforcement and the public about crime and public safety trends and conditions (see Harries, 1995): (1) basic numerical counts on the number of crashes near each pickup spot; (2) a table with data on the number of crashes near each pickup spot; (3) a bar graph with data on the number of crashes near each pickup spot; (4) a line graph with data on the number of crashes near each pickup spot; (5) pie graph with data on the number of crashes near each pickup spot; (6) a hotspot map depicting the relative number of crashes near each pickup spot indicated by gradated colors (green–yellow–orange–red); (7) a hotspot map depicting the relative number of crashes near each pickup spot indicated by gradated grayscale (light gray–medium gray–dark gray–black); (8) a thematic map depicting the relative number of crashes near each pickup spot indicated by streets in gradated colors (green–yellow–orange–red); or (9) a thematic map depicting the relative number of crashes near each pickup spot indicated by streets in gradated grayscale (light gray–medium gray–dark gray–black). For the hotspot maps and thematic maps, risk information was layered upon the type of map that the participant had originally received (satellite map or street map). This meant that no participant that had initially received a satellite map of the pick-up area then randomly received a hotspot map or thematic map layered onto a street map, or vice versa.

After receiving their stimuli, all participants were asked to answer a series of questions that measured their (1) *Perceived Risk* of each of the pick-up points (North, South, East, and West); (2) *Negative Affect* toward each of the pick-up points (North, South, East, and West); (3) *Risky Choice* toward each of the pick-up points (North, South, East, and West); and (4) *Ranking* of the pick-up points (North, South, East, and West). Measures were directly patterned from those used in Van Gelder’s et al. (2009), who used similar measures to examine the role of cognitive and affective decision-making as predictors of risk-based decision-making involving vignettes containing descriptions of risky situations.

*Perceived Risk* measured participants’ cognitive risk processing (i.e., System 2 processing, “risk-as-analysis”) toward each pick-up point. Two items, from Van Gelder’s et al. (2009) and adapted for the current scenario, measured participants’ *perceived risk* as it pertained to risk probability (i.e., “How big are the chances of you being involved in a pedestrian-crash on your way to the North pick-up point?” [1-*very small* to 10-*very big*]) and risk magnitude (i.e., “How serious are the possible consequences of being involved in a pedestrian-crash on your way to the North pick-up point?” [1-*not serious at all* to 10-*very serious*]) concerning getting picked up at each of the pick-up points. The perceived risk score for each pick-up point was obtained by multiplying these two items for a score between 1 and 100 (with higher scores indicating higher cognitive perceptions of risk). The choice to multiply items, rather than averaging them, is in correspondence with the theoretical definition of risk, which combines risk magnitude and probability (Yates and Stone, 1992; Loewenstein and Hsee, 2001; Van Gelder’s et al., 2009).

*Negative Affect* measured participants’ affective risk processing (i.e., System 1 processing, “risk-as-feelings”) toward each pick-up point. Four items, once again from Van Gelder’s et al. (2009) and adapted for the current scenario, measured *Negative Affect* (i.e., “Would you be worried to walk to the North pick-up point?”, “Does walking to the North pick-up point make you feel uncertain?”, “Does walking to the North pick-up point evoke feelings of fear?” and “Does walking to the North pick-up point evoke negative feelings in general?” [1-*not at all* to 10-*very much*]). Cronbach’s alpha of the negative affect scale was 0.83 to 0.92. The composite negative affect score for each pick-up point was obtained by averaging these four items, which resulted in a score between 1 and 10 (with higher scores indicating higher affective perceptions of risk). As the items for *Negative Affect* measured the same construct, rather than items that measured different aspects of it (e.g., risk magnitude and risk probability as distinct elements of *Perceived Risk*), we also chose to create a composite by averaging, rather than multiplying, ratings of these items per Van Gelder’s et al.’s (2009) method.

Two outcome variables related to risk-based decision-making were assessed. The first outcome variable, *Risky Choice*, measured participants’ choices to be picked up at certain pick-up points. *Risky Choice* was measured with two items directly patterned from Van Gelder’s et al. (2009) for the current scenario. The first item asked participants about the likelihood that they would choose to be picked up at each pick up point (i.e., “How likely is it that you would choose to be picked up at the North pick-up point?” [1-*very unlikely* to 10-*very likely*]), and the second item measured certainty (i.e., “How certain are you about your choice to be picked up at the North pick-up point?” [1-*not at all* to 10-*completely*]). *Risky Choice* for each pick-up point was calculated by multiplying participants’ likelihood and certainty scores for each pick-up point, so the scores could range from 1 to 100 (the higher the score, the more likely participants’ would choose to be picked up at that spot). Van Gelder’s et al. (2009) chose to multiply ratings of risk likelihood and risk certainty, as these two combined constitute the known theoretical considerations in risk-based choices. Based on this and Van Gelder’s et al. (2009) past methodological choices, we chose to multiply, rather than average, these items for the *Risky Choice* outcome variable.

The second outcome variable, *Ranking*, asked participants to “Rank the four pick-up points from 1 (most likely to choose to be picked up) to 4 (least likely to choose to be picked up)” (the higher the score/rank, the less likely participants’ were to choose to be picked up picked up at that spot). *Ranking* was calculated using this single item, and it ranged in a score from 1 to 4.

Finally, we wanted to examine how changing the risk context could potentially affect participants’ risk-based decisions. As risk information is often provided for varying geographic distances, such as street-level, neighborhood-level, or city-level, to contextualize and compare types of risk from one place to another (Pridemore, 2005; Deane et al., 2008; McDowall and Loftin, 2009), we wanted to provide some further risk information to contextualize the risk level for the pick-up area. After answering all of the questions noted above, participants were presented with one more piece of information on the risk in their pickup area relative to the city-level risk in Anytown, United States. Regardless of the type of map or risk information they received (or whether they were in the control groups), participants were randomly informed that the 216 crashes in their map extent represent either 5% or 75% of the total crashes within Anytown, United States.

Further data analysis shows that the 216 motor vehicle crashes involving pedestrians within the quarter-mile pick-up area from your location represents (5% or 75%) of total motor vehicle crashes involving pedestrians within Anytown, United States. This means that your location experiences a relatively (small or large) number of pedestrian crashes compared to other areas in Anytown, United States.

Following this additional information, participants were asked to re-rate their *Ranking* and *Risky Choice* for each of the pick-up points, given this additional information, using the same items and method above. Basic demographics, including gender, age, income, education, religion, state of residence, and political affiliation, were collected at the end of the survey.

## Results

### Demographics of Sample

A sample of 800 respondents completed the study. Using G^∗^Power, this sample size was enough for sufficient power for *f* = 0.25 (a medium effect size) for power at 0.95 (*df* = 9 for a 2 × 9 [+2] design), for 20 different groups of participants (i.e., a 2 × 9 [+2] design). The sample was 51.12% male with a *M*_*age*_ = 40.91 years (*SD* = 12.43). The racial breakdown of the sample was that 71.12% identified as White, 6.88% as Black, 10.75% as Hispanic, 5.25% as Asian, and 6.00% as Other or Mixed. The education breakdown was that 9.50% had a high school education, 20.05% had some college, 51.12% had a college degree, 3.12% had some post-graduate study, and 15.75% had a post-graduate degree. Participants also indicated the types of locations in which they had ever lived, and 41.50% indicated they had lived in an urban location for at least a year, 64.38% said they had lived in a suburban location for at least a year, and 24.00% said they had lived in a rural location for at least a year. When asked about how they get to their destinations in their place of residence, 86.38% said they drive regularly, and 24.05% said they walk to destinations regularly. Other demographics, including income, political orientation, and religion, are available upon request. Analyses revealed no significant demographic differences across vignette conditions.

### Effect of Risk Information on Processing and Decision-Making

We first wanted to explore how being presented with some type or form of risk information in relation to crime and public safety (regardless of the specific format) affected participants’ ratings on their *Perceived Risk*, *Negative Affect*, *Risky Choice*, and *Ranking* of each of the pick-up points (North, South, East, and West), compared to those in the control groups with no risk information. We first ran two-way analyses of variance (ANOVAs) for all models and found that all nine types of risk information significantly differed from the control groups for all four measures, and that there were also no significant main, nor interaction, effects for the type of map presented (satellite map and street map) for any of the four measures for the experimental or control groups. Therefore, before using models to examine how the particular type of risk information may have affected participants’ ratings of the measures, we first wanted to explore differences between being presented with risk information versus being presented with no risk information, and we used models that combined all specific types of risk information, as well as both types of maps, and compared them to the control groups. Descriptive statistics, regardless of the type of map, for participants’ ratings of the four above measures for both the control groups and for the experimental groups in which some kind of risk information was presented are found in Tables 1, 2, respectively.

**TABLE 1:** Descriptive statistics for average ratings of measures in control groups (across both satellite and road maps).

Measure	Pick-up point	Mean (*SD*)
Perceived risk (1–100)	North	25.30 (15.23)
	South	25.34 (17.43)
	East	25.19 (15.71)
	West	25.85 (17.36)
Negative affect (1–10)	North	3.87 (2.29)
	South	4.00 (2.40)
	East	3.96 (2.33)
	West	4.01 (2.35)
Risky choice (1–100)	North	37.48 (25.36)
	South	36.51 (27.02)
	East	32.54 (23.70)
	West	29.43 (19.55)
Ranking (1–4)	North	2.82 (0.98)
	South	2.50 (1.18)
	East	2.61 (1.02)
	West	2.58 (0.93)

**TABLE 2:** Descriptive statistics for average ratings of measures when participants received some form of risk data (across both satellite and road maps).

Measure	Pick-up point	Mean (*SD*)
Perceived risk (1–100)	North	36.27 (20.30)
	South	31.66 (16.38)
	East	50.51 (27.31)
	West	18.06 (18.23)
Negative affect (1–10)	North	5.39 (2.37)
	South	4.85 (2.13)
	East	6.44 (2.74)
	West	3.38 (2.28)
Risky choice (1–100)	North	23.48 (22.22)
	South	30.55 (22.60)
	East	15.50 (20.15)
	West	63.10 (32.07)
Ranking (1–4)	North	3.12 (0.71)
	South	2.83 (0.63)
	East	3.54 (0.92)
	West	1.60 (1.08)

For the control groups in which no risk information was presented, one-way ANOVAs showed that participants’ ratings did not significantly differ with regard to any of the four measures for the North, South, East, and West pick-up points across both types of maps (*Perceived Risk*: *F*[3,264] = 0.021, *p* = 0.996; *Negative Affect*: *F*[3,264] = 0.050, *p* = 0.985; *Risky Choice*: *F*[3,264] = 1.60, *p* = 0.191; *Ranking*: *F*[3,264] = 1.17, *p* = 0.321). This assured us that participants had no preconceived notions about choosing or making decisions about the pick-up points even when no risk information was presented, and that responses from our control groups could be considered a baseline for each measure.

We then conducted one-way ANOVAs to see if and how receiving risk information (regardless of the format or type of risk information) significantly affected participants’ ratings on the four measures, compared to the ratings of participants who were in the control groups, regardless of the type of map presented. Tukey HSD *post hoc* tests in cases in which ANOVAs were significant.

Across both types of maps, there were significant main effects for the presentation of risk information on the *Perceived Risk* of the North (*F*[1,798] = 18.59, *p* < 0.0001), South (*F*[1,798] = 4.21, *p* = 0.042), East (*F*[1,798] = 55.80, *p* < 0.0001), and West (*F*[1,798] = 11.30, *p* = 0.0008) pick-up points, compared to the control groups. When participants received risk information, they showed significantly higher ratings on *Perceived Risk* for the North (*t* = 4.31, *p* < 0.0001), South (*t* = 2.05, *p* = 0.042), and East (*t* = 7.47, *p* < 0.0001) pick-up points, compared to those that did not receive any risk information. However, they showed significantly lower ratings on their *Perceived Risk* of the West pick-up point (*t* = −3.36, *p* = 0.001) when participants received risk information, as compared to those that did not receive any risk information. This pattern was consistent for all types of risk information.

A similar pattern was seen for participants’ ratings of their *Negative Affect* toward each pick-up point. Across both types of maps, there were significant main effects for the presentation of risk information on participants’ *Negative Affect* toward the North (*F*[1,798] = 18.59, *p* < 0.0001), South (*F*[1,798] = 4.70, *p* = 0.032), East (*F*[1,798] = 51.51, *p* < 0.0001), and West (*F*[1,798] = 4.73, *p* = 0.029) pick-up points, compared to the control groups. Participants showed significantly higher ratings on their *Negative Affect* toward the North (*t* = 5.04, *p* < 0.0001), South (*t* = 2.17, *p* = 0.032), and East (*t* = 7.18, *p* < 0.0001) pick-up points when they received risk information, as compared to participants that did not receive any risk information. However, participants showed significantly lower ratings on their *Negative Affect* toward the West pick-up point (*t* = −2.18, *p* = 0.030) when receiving risk information, as compared to the control groups.

An opposite, but corresponding, pattern was seen for participants’ *Risky Choice* toward and *Ranking* of each of the pick-up points. Across both types of maps, there were significant main effects for the presentation of risk information on participants’ *Risky Choice* toward and *Ranking* of the North (*Risky Choice*: *F*[1,798] = 23.77, *p* < 0.0001; *Ranking*: *F*[1,798] = 4.12, *p* = 0.044), South (*Risky Choice*: *F*[1,798] = 4.12, *p* = 0.042; *Ranking*: *F*[1,798] = 4.08, *p* = 0.045), East (*Risky Choice*: *F*[1,798] = 42.53, *p* < 0.0001; *Ranking*: *F*[1,798] = 60.78, *p* < 0.0001), and West (*Risky Choice*: *F*[1,798] = 71.35, *p* < 0.0001; *Ranking*: *F*[1,798] = 115.48, *p* < 0.0001) pick-up points, compared to the control groups. Participants were significantly less likely to choose to be picked up at the North (*Risky Choice*: *t* = −4.88, *p* < 0.0001), South (*Risky Choice*: *t* = −2.03, *p* = 0.043), and East (*Risky Choice*: *t* = −6.52, *p* < 0.0001) pick-up points when given risk information, as compared to those that did not receive any risk information. Participants also ranked each of those three pick-up points significantly higher when receiving risk information, meaning that they were significantly less likely to indicate it as a pick-up point they would choose, via their *Ranking* (North: *t* = 2.03, *p* = 0.044; South: *t* = 2.02, *p* = 0.045; and East: *t* = 7.80, *p* < 0.0001). However, they were significantly more likely to rank (*Ranking*: West: *t* = −10.75, *p* < 0.0001) and choose (*Risky Choice*: *t* = 8.45, *p* < 0.0001) to be picked up at the West pick-up point when being presented with risk information, as compared to those that did not receive any risk information.

### Effects for Type of Map × Type of Risk Information on Processing and Decision-Making

Next, we wanted to examine if and how the particular type of risk information may have affected participants’ ratings on *Perceived Risk*, *Negative Affect*, *Risky Choice*, and *Ranking* of each of the pick-up points (North, South, East, and West). We first ran two-way ANOVAs to examine how participants’ ratings on the measures for each pick-up point may have significantly differed from one another due to (1) the type of map (satellite map or street map) or (2) the type of risk information (numerical counts, table, bar graph data, line graph, pie graph, hotspot map in color, hotspot map in grayscale, thematic map in color, and thematic map in grayscale), and their interactions, that participants had received. Responses of participants who were in the control groups, with no form of risk information, were not included in these analyses. Tukey HSD *post hoc* tests were used in cases in which ANOVAs were significant to identify the type or nature of the observed effects. Non-significant main effects for all measures for each pick-up point are found in the Supplementary Tables 1, 2.

For *Perceived Risk*, there were significant interaction effects for the type of risk information and map type on participants’ *Perceived Risk* of being picked up at the North (*F*[8,715] = 3.11, *p* = 0.002), South (*F*[8,715] = 2.57, *p* = 0.009), and East (*F*[8,715] = 3.52, *p* = 0.005) pick-up spots. Similar patterns were seen for the same interaction for all three pick-up points. The *Perceived Risk* of being picked up at the North pick-up point (*M* = 28.58, *SE* = 3.25), South pick-up point (*M* = 19.37, *SE* = 2.61), and East pick-up point (*M* = 35.94, *SE* = 4.58) was significantly lower (at α = 0.05) when participants were presented with hotspot map in color on a satellite map, compared to the other types of risk information (Table 3). However, there was a different significant interaction effect for participants’ *Perceived Risk* of being picked up in the West pick-up spot (*F*[8,715] = 4.11, *p* = 0.001). The *Perceived Risk* of being picked up at the West pick-up spot (*M* = 35.67, *SE* = 3.94) was significantly higher (at α = 0.05) when participants were presented with thematic map in grayscale on a satellite map, compared to the other types of risk information (Table 3).

**TABLE 3:** Ratings of *Perceived Risk* for each pick-up point across types of risk data analysis and type of map (*M*, *SE*) (bold values are those that are significantly different at the *p* < 0.05 level from the italicized values associated with each pick-up point).

Type of risk data analysis	North		South		East		West	
	Road map	Satellite map	Road map	Satellite map	Road map	Satellite map	Road map	Satellite map
Numerical data	*36.68 (2.92)*	*38.96 (2.98)*	*28.53 (2.35)*	*27.80 (3.40)*	*48.36 (3.84)*	*49.13 (3.92)*	*20.06 (2.55)*	*18.13 (2.60)*
Table data	*36.68 (3.02)*	*38.32 (3.02)*	*28.59 (2.43)*	*30.87 (2.43)*	*48.32 (3.97)*	*54.16 (16)*	*15.80 (2.63)*	*20.68 (2.63)*
Bar chart data	*39.21 (3.21)*	*41.82 (3.05)*	*27.39 (2.58)*	*31.67 (2.46)*	*54.56 (4.22)*	*51.47 (4.01)*	*19.15 (2.80)*	*13.40 (2.66)*
Line chart data	*36.67 (3.21)*	*37.16 (3.12)*	*32.97 (2.58)*	*30.11 (2.43)*	*55.08 (4.21)*	*53.82 (3.97)*	*14.97 (2.80)*	*15.42 (2.63)*
Pie chart data	*38.71 (3.13)*	*33.88 (3.05)*	*30.93 (2.51)*	*29.35 (2.46)*	*48.63 (4.11)*	*47.95 (4.01)*	*14.39 (2.72)*	*20.53 (2.66)*
Hotspot map in color	*41.19 (3.29)*	**28.58 (3.25)**	*31.03 (2.65)*	**19.37 (2.61)**	*50.81 (4.33)*	**35.94 (4.58)**	*18.38 (2.87)*	*15.42 (2.83)*
Hotspot map in black and white	*35.18 (3.21)*	*38.00 (3.34)*	*32.85 (2.58)*	*28.08 (2.55)*	*53.97 (4.27)*	*47.54 (4.25)*	*20.72 (2.80)*	*16.80 (2.76)*
Thematic map in color	*40.50 (3.34)*	*35.62 (3.21)*	*28.78 (2.68)*	*27.41 (2.58)*	*47.68 (4.39)*	*47.90 (4.22)*	*18.03 (2.91)*	*16.62 (2.80)*
Thematic map in black and white	*36.45 (3.09)*	*35.33 (3.49)*	*33.95 (2.48)*	*30.97 (2.80)*	*47.95 (4.06)*	*54.94 (4.58)*	*17.05 (2.69)*	**35.67 (3.94)**

For *Negative Affect*, there were significant interaction effects for the type of risk information and map type presented regarding participants on their *Negative Affect* toward the East (*F*[8,715] = 2.36, *p* = 0.017) and West (*F*[8,715] = 2.09, *p* = 0.034) pick-up spots, but not for the North (*F*[8,715] = 1.84, *p* = 0.067) or South (*F*[8,715] = 1.12, *p* = 0.348). The *Negative Affect* toward being picked up at the East pick-up point was significantly higher when participants were presented with a hotspot map in color on a street map (*M* = 7.84, *SE* = 0.44) compared to the other types of risk information (see Table 4). Conversely, participants’ *Negative Affect* toward being picked up at the West pick-up point was significantly higher when participants were presented with thematic map in grayscale on a satellite map (*M* = 4.97, *SE* = 0.39), compared to the other types of risk information (Table 4).

**TABLE 4:** Ratings of *Negative Affect* for each pick-up point across types of risk data analysis and type of map (*M*, *SE*) (bold values are those that are significantly different at the *p* < 0.05 level from the italicized values associated with each pick-up point).

Type of risk data analysis	North		South		East		West	
	Road map	Satellite map	Road map	Satellite map	Road map	Satellite map	Road map	Satellite map
Numerical data	5.55 (0.34)	5.56 (0.35)	4.78 (0.31)	4.66 (0.32)	*6.59 (0.39)*	*6.13 (0.40)*	*3.68 (0.33)*	*3.69 (0.33)*
Table data	4.88 (0.35)	5.28 (0.35)	4.06 (0.32)	4.80 (0.32)	*6.01 (0.41)*	*6.32 (0.41)*	*3.15 (0.34)*	*3.13 (0.34)*
Bar Chart Data	5.59 (0.38)	5.19 (0.36)	4.23 (0.34)	4.76 (0.32)	*6.47 (0.43)*	*6.82 (0.41)*	*3.03 (0.36)*	*3.19 (0.34)*
Line chart data	5.60 (0.38)	5.86 (0.35)	4.41 (0.34)	4.86 (0.32)	*6.49 (0.43)*	*6.48 (0.41)*	*3.28 (0.36)*	*3.43 (0.34)*
Pie chart data	5.31 (0.37)	5.07 (0.36)	4.69 (0.33)	4.16 (0.32)	*5.80 (0.42)*	*6.36 (0.41)*	*3.09 (0.35)*	*3.11 (0.34)*
Hotspot map in color	5.01 (0.39)	4.83 (0.38)	4.73 (0.35)	4.16 (0.344)	**7.84 (0.44)**	*6.14 (0.44)*	*3.52 (0.37)*	*3.11 (0.36)*
Hotspot map in black and white	4.89 (0.37)	4.91 (0.37)	4.11 (0.34)	4.25 (0.34)	*6.50 (0.43)*	*6.41 (0.43)*	*3.65 (0.36)*	*3.15 (0.35)*
Thematic map in color	5.56 (0.38)	5.13 (0.38)	4.46 (0.35)	4.22 (0.34)	*6.05 (0.45)*	*5.74 (0.43)*	*3.69 (0.37)*	*3.70 (0.34)*
Thematic map in black and white	5.63 (0.36)	5.17 (0.41)	4.46 (0.33)	4.84 (0.37)	*6.10 (0.42)*	*6.30 (0.47)*	*3.33 (0.34)*	**4.97 (0.39)**

For *Risky Choice*, there was a significant interaction effect for the type of risk information and map type presented regarding participants on their *Risky Choice* toward only the West pick-up spot (*F*[8,715] = 2.05, *p* = 0.039), and not for North (*F*[8,715] = 1.22, *p* = 0.285), South (*F*[8,715] = 1.31, *p* = 0.237), or East (*F*[8,715] = 1.51, *p* = 0.150). Participants were significantly less likely to choose to be picked up at the West pick-up point, via their *Risky Choice* ratings, when they were presented with thematic map in grayscale on a satellite map (*M* = 38.45, *SE* = 3.74), compared to the other types of risk information (Table 5).

**TABLE 5:** Ratings of *Risky Choice* for each pick-up point across types of risk data analysis and type of map (*M*, *SE*) (bold values are those that are significantly different at the *p* < 0.05 level from the italicized values associated with each pick-up point).

Type of risk data analysis	North		South		East		West	
	Road map	Satellite map	Road map	Satellite map	Road map	Satellite map	Road map	Satellite map
Numerical data	26.61 (3.14)	19.29 (3.21)	32.36 (3.25)	33.00 (3.33)	16.21 (2.89)	17.13 (2.96)	*61.40 (4.53)*	*69.31 (4.63)*
Table data	25.64 (3.24)	18.18 (3.24)	33.11 (3.36)	31.89 (3.36)	18.55 (2.99)	14.39 (2.99)	*66.18 (4.69)*	*64.07 (4.69)*
Bar chart data	18.54 (3.24)	26.37 (3.28)	27.49 (3.57)	27.47 (3.40)	13.44 (3.18)	18.69 (3.03)	*65.77 (4.98)*	*65.84 (4.74)*
Line chart data	21.31 (3.44)	21.93 (3.24)	29.85 (3.57)	25.57 (3.36)	14.08 (3.18)	12.21 (2.99)	*63.82 (4.98)*	*64.16 (4.69)*
Pie chart data	17.66 (3.35)	18.00 (3.28)	25.10 (3.48)	29.42 (3.40)	12.22 (3.10)	11.84 (3.03)	*69.34 (4.85)*	*68.40 (4.74)*
Hotspot map in color	26.41 (3.49)	26.05 (3.49)	27.14 (3.67)	30.18 (3.61)	15.11 (3.26)	17.55 (3.22)	*57.43 (5.11)*	*62.00 (5.04)*
Hotspot map in black and white	25.38 (3.44)	22.20 (3.400)	32.36 (3.57)	32.23 (3.53)	18.77 (3.18)	14.30 (3.14)	*61.05 (4.98)*	*63.93 (4.91)*
Thematic map in color	24.08 (3.58)	26.82 (3.44)	35.19 (3.53)	35.10 (3.57)	16.17 (3.31)	16.51 (3.18)	*62.83 (5.18)*	*62.74 (4.98)*
Thematic map in black and white	22.07 (3.32)	27.06 (3.74)	32.12 (3.44)	29.94 (3.88)	18.67 (3.06)	18.79 (3.45)	*58.62 (4.80)*	**39.79 (5.41)**

For *Ranking*, there was a significant interaction effect for the type of risk information and map type regarding participants’ *Ranking* of the West pick-up spot (*F*[8,715] = 2.05, *p* = 0.039), but not for North (*F*[8,715] = 0.18, *p* = 0.668), South (*F*[8,715] = 1.71, *p* = 0.094), or East (*F*[8,715] = 1.51, *p* = 0.150). Participants were significantly less likely to rank the West pick-up point as a pick-up point they would choose, via their *Ranking*, when they were presented with thematic map in grayscale on a satellite map (*M* = 2.70, *SE* = 0.18), compared to the other types of risk information (Table 6).

**TABLE 6:** Ratings of *Ranking* for each pick-up point across types of risk data analysis and type of map (*M*, *SE*) (bold values are those that are significantly different at the *p* < 0.05 level from the italicized values associated with each pick-up point).

Pick-up point	North		South		East		West	
	Road map	Satellite map	Road map	Satellite map	Road map	Satellite map	Road map	Satellite map
Numerical data	3.03 (0.10)	3.14 (0.10)	2.81 (0.09)	2.80 (0.09)	3.49 (0.13)	3.47 (0.13)	*1.67 (0.15)*	*1.51 (0.15)*
Table data	3.11 (0.10)	3.13 (0.10)	3.20 (0.09)	3.00 (0.09)	3.55 (0.14)	3.74 (0.14)	*1.37 (0.16)*	*1.41 (0.16)*
Bar chart data	3.12 (0.11)	3.22 (0.11)	2.70 (0.10)	2.95 (0.09)	3.72 (0.14)	3.41 (0.14)	*1.35 (0.16)*	*1.42 (0.16)*
Line chart data	3.24 (0.11)	3.13 (0.11)	2.97 (0.10)	3.05 (0.10)	3.44 (0.14)	3.64 (0.14)	*1.54 (0.17)*	*1.47 (0.16)*
Pie chart data	3.19 (0.11)	3.22 (0.10)	2.78 (0.10)	2.75 (0.10)	3.68 (0.14)	3.41 (0.14)	*1.53 (0.16)*	*1.46 (0.16)*
Hotspot map in color	2.94 (0.11)	2.98 (0.11)	3.14 (0.10)	2.94 (0.11)	3.49 (0.15)	3.56 (0.15)	*1.67 (0.16)*	*1.56 (0.17)*
Hotspot map in black and white	2.93 (0.11)	2.98 (0.11)	2.72 (0.10)	2.75 (0.10)	3.55 (0.14)	3.75 (0.14)	*1.76 (0.17)*	*1.50 (0.16)*
Thematic map in color	2.92 (0.11)	3.16 (0.11)	3.11 (0.10)	2.84 (0.10)	3.38 (0.14)	3.37 (0.14)	*1.67 (0.17)*	*1.75 (0.17)*
Thematic map in black and white	2.91 (0.11)	3.12 (0.12)	2.73 (0.10)	2.76 (0.11)	3.47 (0.14)	3.50 (0.15)	*1.79 (0.16)*	**2.70 (0.18)**

### Association Between Cognitive and Affective Processing and Risk-Based Decisions

Next, we examined if and how *Perceived Risk* of and *Negative Affect* toward each pick-up spot predicted participants’ *Risky Choice* toward and *Ranking* of each pick-up spot. Given the fact that independent and dependent measures were ordinal in nature, we used binomial logistic regression models and chose to dichotomize participants’ responses for their *Risky Choice* toward each pick-up point and *Ranking* of each pick-up point for this analysis (dependent measures). *Ranking* was dichotomized as a dummy variable by classifying two groups: low likelihood of being chosen as a pick-up spot (participants’ *Ranking* of the spot as either 3 or 4; coded as 0) and high likelihood of being chosen as a pick-up spot (participants’ *Ranking* of the spot as either 1 or 2; coded as 1). *Risky Choice* was also dichotomized as a dummy variable by classifying two groups: low likelihood of being picked up at this spot (participants’ *Risky Choice* rating of the spot from 1 to 50; coded as 0) and high likelihood of being picked up at this spot (participants’ *Risky Choice* rating of the spot from 51 to 100; coded as 1). Similarly, for these models, we also dichotomized participants’ *Perceived Risk* of and *Negative Affect* ratings of each pick-up point (the independent variables), as they were also ordinal in nature. *Perceived Risk* was dichotomized as a dummy variable by classifying two groups: low perceived risk of the pick-up spot (participants’ *Perceived Risk* rating of the spot from 1 to 50; coded as 0) and high perceived risk of the pick-up spot (participants’ *Perceived Risk* rating of the spot from 51 to 100; coded as 1). *Negative Affect* was also dichotomized as a dummy variable by classifying two groups: low negative affect toward the pick-up spot (participants’ *Negative Affect* rating of the spot from 1 to 5; coded as 0) and high negative affect toward the pick-up spot (participants’ *Negative Affect* rating of the spot from 6 to 10; coded as 1).

For participants in the control groups who did not receive any risk information, high *Perceived Risk* and high *Negative Affect* did not significantly predict either participants’ likelihood of being picked up at any of the four pick-up points (*Risky Choice*) or the likelihood of them choosing any of the pickup points (*Ranking*) (see Supplementary Tables 3, 4). When participants received any type of risk information, their likelihood of being picked up at any of the pick-up points (*Risky Choice*) was significantly predicted by their high *Perceived Risk* of and high *Negative Affect* toward all four spots (Table 7). Participants’ likelihood of choosing the North and South pick-up points (*Ranking*) was only significantly predicted by their high *Perceived Risk* of, but not their high *Negative Affect* toward, those two pick-up spots when receiving some form of risk information (Table 8). Yet, both participants’ high *Perceived Risk* of and high *Negative Affect* toward the West and East pick-up points significantly predicted their likelihood of choosing to be picked-up at those two locations (*Ranking*) (Table 8).

**TABLE 7:** Binary logistic regression results showing the predictive power of high *Perceived Risk* and high *Negative Affect* toward the high likelihood of being picked up at each spot (*Risky Choice*) when participants received some form of risk analysis data.

Measure	Pick-up point	*Coefficient*	*Odds ratio*	*SE*	*Z*	*p*-value
High perceived risk	High likelihood of being picked up at the North	−1.97	7.17	0.308	−6.41	<0.0001
	High likelihood of being picked up at the South	−0.689	1.99	0.285	−2.42	0.016
	High likelihood of being picked up at the East	−1.20	3.32	0.288	−4.17	<0.0001
	High likelihood of being picked up at the West	−3.58	35.87	0.349	−10.25	<0.0001
High negative affect	High likelihood of being picked up at the North	−2.12	8.33	0.206	−10.27	<0.0001
	High likelihood of being picked up at the South	−2.25	9.49	0.319	−7.05	<0.0001
	High likelihood of being picked up at the East	−1.67	5.31	0.222	−7.50	<0.0001
	High likelihood of being picked up at the West	−2.93	18.73	0.318	−9.20	<0.0001

**TABLE 8:** Binary logistic regression results showing the predictive power of high *Perceived Risk* and high *Negative Affect* toward the high likelihood of being chosen as the pick-up spot (*Ranking*) when participants received some form of risk analysis data.

Measure	Pick-up point	*Coefficient*	*Odds ratio*	*SE*	*Z*	*p*-value
High perceived risk	High likelihood of the North being chosen as the pick-up spot	−1.10	3.00	0.249	−4.40	<0.0001
	High likelihood of the South being chosen as the pick-up spot	−1.16	3.19	0.303	−3.82	<0.0001
	High likelihood of the East being chosen as the pick-up spot	−1.50	4.48	0.243	−6.16	<0.0001
	High likelihood of the West being chosen as the pick-up spot	−2.03	7.61	0.305	−6.67	<0.0001
High negative affect	High likelihood of the North being chosen as the pick-up spot	−0.274	1.32	0.190	−1.44	0.151
	High likelihood of the South being chosen as the pick-up spot	−0.257	1.29	0.207	−1.24	0.215
	High likelihood of the East being chosen as the pick-up spot	−0.515	1.67	0.217	−2.36	0.018
	High likelihood of the West being chosen as the pick-up spot	−1.57	4.81	0.204	−7.70	<0.0001

For those receiving some sort of risk information, the Spearman’s Rank correlations (*r*_*s*_) and their significance levels between their *Perceived Risk* of and *Negative Affect* toward each pick-up spot and the two outcome variables are found in Table 9. This type of correlation was used due to the ordinal nature of participants’ ratings. Correlation coefficients show moderate relationships between participants’ *Perceived Risk* and *Negative Affect* ratings toward their *Risky Choice* and *Ranking* of the West pick-up spot, and weak relationships between these variables for the other pick-up spots.

**TABLE 9:** Spearman’s rank correlations (*r*_*s*_) between *Perceived Risk* and *Negative Affect* and participants’ *Risky Choice* and *Ranking* for each pick-up spot.

Outcome	North		South		East		West	
	Perceived risk	Negative affect	Perceived risk	Negative affect	Perceived risk	Negative affect	Perceived risk	Negative affect
Risky choice	−0.144 (p = 0.002)	−0.173 (p < 0.0001)	−0.048 (p = 0.180)	−0.073 (p = 0.040)	−0.353 (p < 0.0001)	−0.329 (p < 0.0001)	−0.420 (p < 0.0001)	−0.589 (p < 0.0001)
Ranking	0.210 (p < 0.0001)	0.207 (p < 0.0001)	0.075 (p = 0.035)	0.051 (p = 0.155)	0.345 (p < 0.0001)	0.268 (p < 0.0001)	0.432 (p < 0.0001)	0.609 (p < 0.0001)

### Effect of City-Level Risk Context on Risk-Based Decision-Making

Finally, three-way ANOVAs were run (with type of risk information, type of map, and the % of crashes in the pick-up area as independent variables) to see if participants’ ratings on *Risky Choice* and *Ranking* changed for any of the pick-up points when presented with information that the crashes in the pick-up radius made up either 5% or 75% of crashes across the entirety of Anytown, United States (as well as the interactions with the other two independent variables). Participants who were in the control groups were also included in these analyses, as they also received information on how the percentage of crashes in the pick-up radius compared to the entirety of Anytown, United States, and its type of risk information was coded as none.

There were no significant differences found between participants’ original ratings of *Risky Choice* and *Ranking* for any of the pick-up points, regardless of the type of risk information or map received, and their ratings when presented with either the information that the crashes in the pick-up radius made up either 5% or 75% of crashes across the entirety of Anytown, United States. Non-significant main and interaction effects are found in the Supplementary Table 5.

## Discussion

This current study, using a multi-factorial vignette-based survey experiment with a sample of the general public, investigates if and how common types of risk information used to communicate public safety and crime conditions elicit dual-process risk information processing in risk-based decisions, and if such processing or decision-making differs depending on the risk level, context, or the type or format of risk information communicated. In summary, this experiment found that risk information on public safety does in fact elicit dual-process risk information processing and influences risk-based decision-making for all levels of risk, as compared to information processing and decision-making with no risk information. As hypothesized, both affective and cognitive processing were significant predictors of different risk-based decisions, regardless of the risk level or type of risk information examined in the current study. When it came to specific types of risk information, hotspot maps in color on a satellite map, compared to the other types of risk information, elicited significantly lower levels of cognitive risk processing when considering pick-up spots that represented any risk level higher than the lowest level of risk. For the pick-up spot representing the lowest risk level, thematic maps in grayscale on a satellite map elicited higher levels of both cognitive and affective risk processing, and, correspondingly, participants were significantly less likely to choose or rank to be picked up at that spot when receiving that type of risk information. Even when varying degrees of city-level risk were provided as context to participants, risk-based decision-making did not significantly change whatsoever for any type of risk information or pick-up spot.

Results suggest three main takeaways with regard to how risk information may affect dual-process risk information processing and risk-based decision-making related to public safety when considering varying levels of relative risk.

First, our findings provide first-of-its-kind data showing that members of the general public, as consumers of risk information used to communicate public safety and crime conditions, process and make decisions surrounding such information using the dual-process approach. These results align with existing literature that shows that, if presented with risk information, both cognitive and affective modes of risk information processing influence recognition and risk-based decision-making (Ferreira et al., 2006; Evans, 2008; Van Gelder’s et al., 2009). This work also extends the results of previous work specifically to common types of risk information related to crime and public safety. In this study, cognitive and affective processing was not elicited and neither mode of processing predicted risk-based choices, for any level of risk, without some form of risk information present. Thus, communicating risk information used in crime and public safety appears to alter the modes in which consumers process and make decisions via the dual-process approach.

Second, our results suggest that basic forms of risk information used to communicate public safety and crime conditions appear to create an almost “binary-type” distinction for its consumers in which the choice or option representing the lowest risk level is often treated differently in risk processing and decision-making, as compared to other higher levels of risk. The lowest-risk level, not the highest-risk level, may serve as the point of comparison for risk-based decision-making.

For example, the lowest risk option, rather than the highest risk as hypothesized, appeared to act as a “reference point” in several areas of processing and decision-making. Participants showed no differences in processing or making decisions about the pick-up points when no risk was presented. Yet, when presented with some type of risk information, participants unsurprisingly showed significantly higher levels of cognitive and affective risk processing when considering the North, South, or East pick-up points, and they were significantly less likely to choose or rank those locations as their pick-up points. Thus by providing some type or level of risk, participants perceived all three of those locations as both cognitively and affectively riskier than when no risk information was provided, and they were less likely to choose to be picked up there.

However, this pattern did not appear to extend to their processing and choices surrounding the pick-up spot representing the lowest risk level. When presented with some type of risk information, participants were *more* likely to choose or rank to be picked up at the West pick-up point, which represented the lowest level of possible risk, compared to when no risk information was presented. This result itself was not surprising, as participants were forced to choose to be picked up at one of the four spots, and all options had some level of risk present. Participants, logically, would likely choose the least risky option when provided risk information on the pick-up area.

Yet, what was interesting was that *lower* levels of cognitive and affective risk processing were elicited for the West pick-up point when presented with some form of risk information, as compared to when no risk information was presented for that location. This result, at first perplexing, suggests that participants literally perceived *less* cognitive and affective risk toward the West pick-up point when *some* level of risk was communicated, as compared to when *no* risk was communicated. Why would adding risk information lead to *lower* levels of cognitive and affective risk processing for the least risky option, as compared to the other three higher risk options?

We suggest that consumers of risk information may be exhibiting *lower* levels of cognitive and affective risk processing toward the lowest risk option in this context because, during risk information processing of public safety and crime data, they are comparing its risk level as relative to the other higher risk options. By using the lowest risk option as their comparison point during risk information-processing, they may actually perceive *less* cognitive and affective risk than they would when no risk information is provided because their reference point in decision-making, due to the risk information, has changed in order to discount, or disapprove, higher risk options and choose, or approve of, the lowest risk option. Ultimately, individuals may put emphasis and focus on the lowest-risk option when making risk-based decisions, and then potentially compare it to all other possible higher risk options, regardless if other options are just slightly more risky or considerably more risky. Indeed, beyond identifying the lowest risk option, participants appeared to not discern varying or nuanced levels of risk (i.e., 43, 67, or 85 pedestrian crashes) during cognitive and affective processing.

Similarly, we saw that affective processing was strongest and more important for risk-based decisions involving the lowest risk option, relative to other higher risk options. Participants’ rankings of the North and South as pick-up points they would choose were only predicted by cognitive, and not by affective, risk processing. Although participants’ ranking of the East pick-up spot was also significantly predicted by affective processing, their ranking of the lowest risk option, the West, was much more highly significantly predicted by affective risk processing. Participants’ choices to be picked up at the West pick-up spot, as compared to the other three pick-up spots, was more strongly predicted by affective processing as well. Further, the moderate association between participants’ both cognitive and affective risk processing and their choice or rank of pick-up points was strongest for the West as the lowest risk option, while the other three pick-up options showed weak associations between both modes of risk processing and risk-based decisions. These results appear to also suggest a potential “divide” in risk information processing, and that consumers may be in fact comparing two black-and-white (i.e., binary) options (i.e., lowest risk option versus any higher risk options), rather than processing four separate options.

The potential “binary-type” distinction for consumers in which the lowest risk option may be treated differently in risk processing and decision-making, relative to other higher risk options, was contrary to our hypothesis. It also conflicts with large bodies of existing work that show that the information processing of numerous types or areas of risk information, ranging from health (Griffin et al., 1999), the environment (Marx et al., 2007), the buying of risky products (Diamond, 1988), food choices (You and Ju, 2017), gambling (Nygren, 1998), to drunk driving (Gibbons et al., 2009), focus on or emphasize high-risk options in order to make risk-based decisions. Further, also contrary to our hypothesis and previous literature, affective processing (i.e., “risk-as-feelings”) was more important and predictive of risk-based decision-making involving the lowest risk level. Indeed, previous work has found that affective processing may be most important for high-risk related decision-making, influenced by fear, and that high levels of affective processing may cloud cognitive evaluations of risk for higher risk situations (Loewenstein and Hsee, 2001). Although replications of these results and further studies are needed, this study suggests that risk information communicating crime and public safety conditions may be processed differently from other types of risk information, such as health, environmental, or financial risk information, when making risk-based decisions.

Third, this research provides the first-of-its-kind data showing that risk information processing, and its effect, on decision-making is not “one size fits all” (Carter et al., 2017). Previous literature has postulated that processing of risk information on crime and public safety may differ depending on the type or format of risk information, such as maps, graphs, or tables, the level of risk being weighed, or the potential consequences of related decisions (Harries, 1995; Lundgren and McMakin, 2018). Indeed, the current study found that risk information significantly influences risk information processing and decision-making in distinctive ways depending on the type of risk information and the level of risk being weighed. Dual-process approaches, while coexisting in risk recognition and decision-making, do appear to respond to different types of risk information or risk-based characteristics (Lerner and Keltner, 2001; Hodgkinson and Clarke, 2007).

**FIGURE 3:**
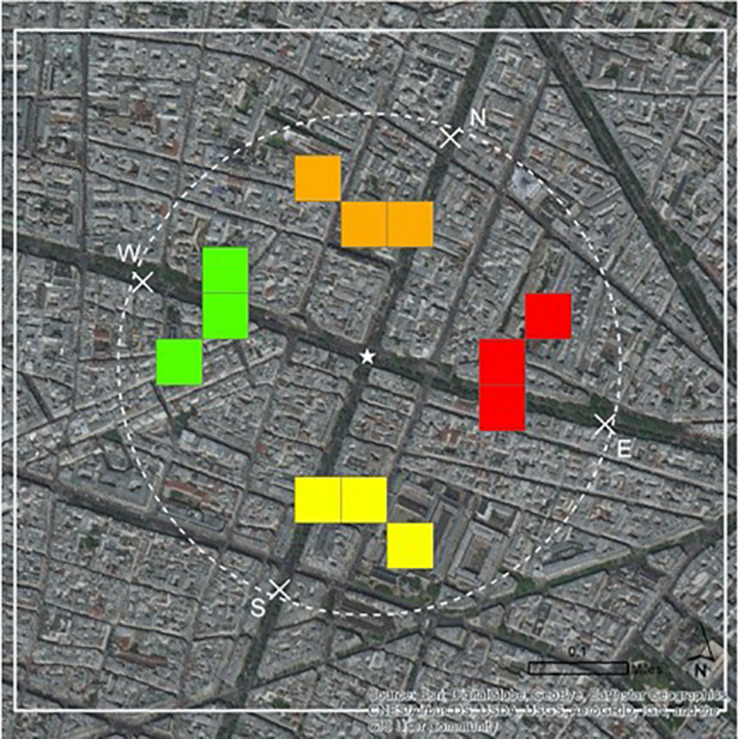
Risk information: hotspot map in color of pick-up area on satellite map.

When it came to specific types of risk information, it is important to point out that, in general, results showed that most types of risk information were processed in ways not significantly different from one another. However, as hypothesized, increased affective processing was observed for some visual forms of risk information, but not for all types of maps or levels of risk (Slovic et al., 2005; Allen and Thomas, 2011; Thompson and Morsanyi, 2012). Yet, contrary to our hypothesis and previous work, cognitive processing was no more elicited for risk information involving probability or numbers, such as graphs, tables, or numerical counts, compared to other types of risk information (Slovic et al., 2005). In fact, maps were shown to be the only type of risk information that influenced cognitive risk processing. Hotspot maps in color on a satellite map (see Figure 3 for stimuli) elicited significantly lower levels of cognitive risk for the North, South, and East pick-up spots, but not for the West (i.e., the lowest risk option). What is it about color hotspots on a satellite map, compared to other risk information, that mitigates cognitive risk processing for the three higher risk options?

We believe that lower levels of cognitive risk processing, in response to the characteristics of this map (colored hotspots on a satellite map), may indicate that participants are able to more efficiently process, identify, and potentially disregard the higher-risk options using this map, perhaps because of the visually apparent spatial contexts or similarities across pick-up spots derived from the satellite imagery, as compared to when using other forms of risk information. As such, they may be in some way more comfortable with their perceptions of these higher-risk options when using this map. Indeed, maps that have clearer, distinctive, and more recognizable characteristics to the map reader, such as using colors, have been thought to be easier to read and understand (Harries, 1995; Chainey and Tompson, 2008). Satellite maps may also offer more detailed factors of the environment for risk-based comparisons (that introduce uncontrolled-for preconceptions that alter judgments), while street maps offer only road-bed representations of a location and minimal context for a location’s characteristics, such as landmarks or vegetation.

**FIGURE 4:**
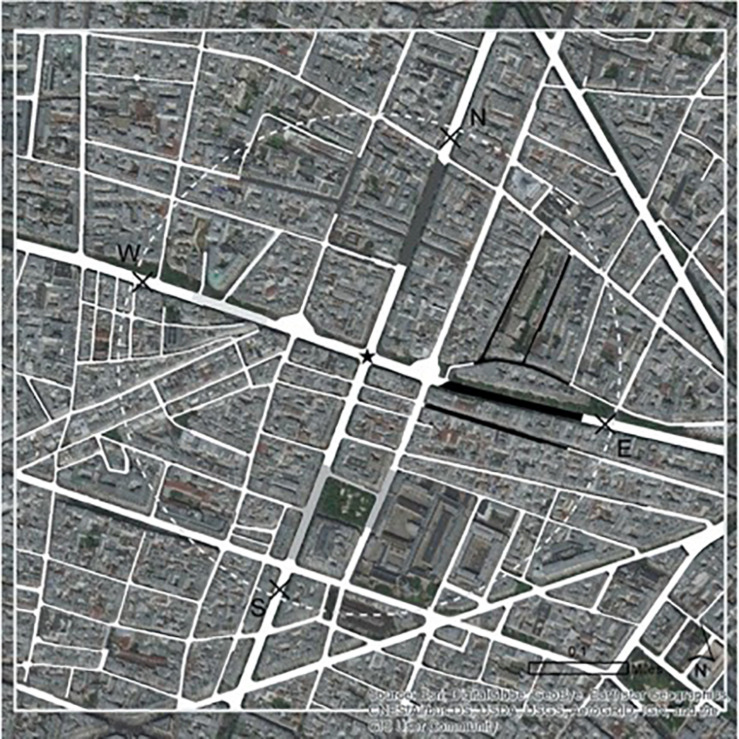
Risk information: thematic map in grayscale of pick-up area on satellite map.

Other results are suggestive of similar reasoning. Thematic maps in grayscale on a satellite map (see Figure 4 for stimuli), compared to the other types of risk information, were found to elicit higher levels of cognitive risk toward the lowest-risk option. Some thematic maps use shading to indicate risk levels, which have been known to be effective at labeling maps and signifying trends in public safety and crime contexts (Eck et al., 2000; Chainey and Tompson, 2008; Weisburd et al., 2012). However, in the current situation, it is possible that the current map’s characteristics may be harder to read and understand and do not provide clear or useful risk communication. Grayscale shading may not be clear or distinctive enough, especially against a satellite map, which has more color ranges and textures than the street map, for consumers to efficiently process, identify, and choose the lowest-risk option. Correspondingly, they may be uncomfortable or uneasy with their level of risk comprehension and feel uncertain whether they are choosing the least risky option.

As fear and discomfort are often at work when higher levels of affective risk processing are exerted (Allen and Thomas, 2011; Thompson and Morsanyi, 2012), it is unsurprising that participants who received the thematic map in grayscale on a satellite map were also found to elicit higher levels of affective risk processing in the same context, and correspondingly, were significantly *less* likely to both choose and rank to be picked up at the lowest-risk option. These patterns were not found for any of the higher risk pick-up points. Thus, findings suggest that participants may not trust their “gut feeling” when risk information is not distinctive or clear enough for effective risk information processing, and that consumers of such information may fear making the wrong risk-based choice (Kruglanski and Orehek, 2007; Evans, 2008). As such, this work may also be relevant to research on the public’s fear of crime, since notions related to cognitive and affective processing have been used to interpret people’s perceived risk and fear of crime in previous literature (e.g., Gabriel and Greve, 2003; Rader, 2004). There are many ripe areas of future inquiry that may look at how risk information interacts with emotional, cognitive, and behavioral components of the public’s fear of being victimized or involved in risk situations (Rader, 2004).

This research does have its limitations. Multi-factorial, vignette-based experiments are commonly used research designs in criminal justice, criminology, and psychology research (i.e., Berryessa, 2017, 2018; Berryessa and Lively, 2019; Berryessa and Krenzer, 2020). Yet, although they are helpful in isolating the effects of independent variables and reducing social desirability, are still less ecologically valid than other types of research designs, and it is unknown if and how these results would extend to real-life contexts involving risk information and decision-making involving crime and public safety (Auspurg and Hinz, 2014). Thus, future work should look to replicate and extend this type of inquiry to different, more ecologically valid research designs and methodologies.

Second, Amazon’s MTurk, although known to produce reliable and robust data from its research samples (Buhrmester et al., 2011; Rouse, 2015), is limited in its sample frame, in that participants self-select into the service and to participate in the survey. Thus, this study’s sample is a non-probability sample; therefore, results from such a sample may be biased by self-selection into the study, and they are by no means representative of the U.S. population. MTurk workers are more likely to be younger, highly educated, and have higher incomes as compared to the average member of the U.S. workforce (Barger et al., 2011). In this study, male participants also appear to be slightly overrepresented, and those with post-graduate degree are overrepresented in the current study’s sample as compared to the general population.

It is true that probability samples, which are the gold standard in survey research because they allow for descriptive and causal inference, are known to provide the most accurate and robust results in empirical work (MacInnis et al., 2018). Yet there are several reasons to suggest that results from non-probability samples, such as these, may also be robust. Experimental methods such as those used in this study, and the lack of demographic differences between conditions, do help to mitigate issues involving representativeness of the research sample (Keppel and Wickens, 2004). Additionally, in application, probability sampling is often marred by coverage, selection, and non-response errors; thus, data from non-probability samples actually are often of comparable data and inferential quality as compared to those from probability samples (Groves and Lyberg, 2010). Indeed, Bartneck et al. (2015) also found that results from MTurk samples most often do not significantly differ from those using samples from survey firms or student populations.

Further, we used calculated composite measures of *Perceived Risk* and *Risky Choice* by multiplying, respectively, the items designed to measure each constructs, but we also calculated the composite measure for *Negative Affect* by averaging its four items. Although these measures and approach were based on the method of Van Gelder’s et al. (2009), it is possible that this study’s results may be affected by our choice to calculate composite measures in different ways, and participants’ responses may have varied if a different approach was applied. As different measures are defined by different scales and dimensions, results may potentially vary if a different approach was chosen to compute these composite measures. Thus, future work should use different approaches, measures, and methodological choices to study these issues to see if the current results hold in different research designs. For example, perhaps a future study should calculate composite measures from averaging the items, which would also allow computing the measures of dispersion between all the items for each composite measure.

Additionally, the fact that we used scales that ranged from 1 to 10 to collect participants’ responses, which gave participants a wide range of options to choose in their responses, may have affected the results and analysis in this paper. We therefore also suggest replication using a smaller (i.e., 1–4) scale for participants’ responses. We also do not know the amount of time spent on each question and how processing time may have differed depending on the pick-up point or processing style. Future work should measure processing times and see if cognitive processing shows longer processing times as compared to affective processing. We also did not collect information about whether participants had previously suffered a pedestrian-automobile crash, which feasibly could have affected their responses. Future work should control for this demographic experience.

Finally, although our results suggest that participants may exhibit a “binary-type” distinction between the lowest risk level and areas with higher risk in risk information processing and decision-making, we also caution that these results may have been influenced by the way in which the research experiment was designed (i.e., a choice of four pick-up points, at one point in time, with explicit, numerical risk levels that differed equally from each other). Of course, people’s processing of risk information is likely to be much more complex in real-case scenarios in which multiple sources of risk information, including previous experiences, interact with each other across many different choices and may vary across time. Thus, these findings do not necessarily mean that risk information processing does indeed function in the real world in the ways observed here. Thus, future work is needed to study these issues with various materials, methods, and research designs before any conclusions can be made.

Nonetheless, this research has several implications for communicating risk information on crime and public safety and its consumers (Cope, 2004). Sir Robert Peel believed that law enforcement’s collection and communication of different types of risk information has two purposes in aiding risk-based decision-making: both to inform the general public of crime and public safety conditions and to aid law enforcement in allocating resources and crafting strategies (Gaines et al., 1999; O’Shea and Nicholls, 2003).

In relation to the general public, maps, graphs, and other police-provided crime and public safety data can help members of the public make real-time risk-based decisions on what areas of a neighborhood or city to frequent or avoid (Bonkiewicz, 2015; Davis et al., 2015; Wheeler, 2016; Roberts, 2018). As the general public appears to exhibit dual-process information processing in relation to these kinds of risk information, this study suggests some ways in which effective communication of risk information by law enforcement can be framed or formatted in order to aid the public in making risk-based decisions in their everyday lives. For example, risk related to crime and public safety, such as the number of assaults, pedestrian-crashes, or robberies occurring in different areas of a city, may be best communicated by highlighting or emphasizing areas or options involving low risk, rather than high risk. Further, maps appear to be helpful to the public in monitoring trends and visualizing data in related decision-making (Guilfoyle, 2016; Wheeler, 2016). Yet, akin to previous literature, if maps are indeed used, they need to be clear, include distinctive attributes, such as colors, and include more detailed renditions of a location to allow for efficient and effective risk processing and decision-making. Maps that lack distinctive features and of which colors or lines are not easy to understand should be avoided, as they appear to potentially cause uncertainty in risk-based decision-making related to crime and public safety conditions (Harries, 1995; Eck et al., 2000; Chainey and Tompson, 2008; Weisburd et al., 2012). Maps with distinct binary categories, such as “lowest-risk” versus “everywhere else,” may be also more actionable and thus preferable.

In relation to law enforcement, this study furthers our understanding of how risk information should be integrated into modern-day information-based police strategies and operations (Reuland, 1997; Coldren et al., 2013; Randol, 2014; Dağlar and Argun, 2016), and provides areas for future research. Although it is unknown how the current results may extend to or differ from a police officer sample, there is some existing research to suggest that members of police departments also exhibit dual-process risk processing in risk-based decision-making, albeit not related to the types of risk-information examined in this study (Sztajnkrycer et al., 2010; Brown and Daus, 2015; Hine et al., 2018). For example, Brown and Daus (2015) found that a sample of 127 police officers exhibited both cognitive and affective processing in response to risk-related work scenarios, such as responding to a hypothetical domestic violence situation, in relation to their decision to shoot a threatening suspect. Indeed, Hine et al. (2018) found officers show dual-process risk processing related to the use of force.

Although potentially much different from the risk processing examined in this study, these studies at least suggest that members of the policing profession sometimes use the dual-process approach to risk processing in on-the-job risk-based decision-making. Juxtaposed with the current findings, it is probable that the various types and formats of crime bulletins and intel products produced by in-house analysts for dissemination to police officers could differentially influence their risk perceptions or deployment strategies above and beyond what the empirical risks might otherwise necessitate. Future work should use a police officer sample with the current research design and materials to see if and how risk information processing and decision-making may differ from the general public. However, if police do in fact exhibit risk processing in similar ways to the public, the current study may help to provide at least cursory directions regarding what types of risk information may be most useful and effective for members of law enforcement in visualizing trends in crime and public safety, making risk-based decisions on the job, or deploying resources, personnel, or strategies to certain locations (Guilfoyle, 2016; Wheeler, 2016). For example, Harries (1995) suggests that patrol officers should be provided the most easily accessible, user-friendly, and detailed data related to their patrol areas in order to best walk their beats. These data suggest that certain characteristics of maps may best communicate risk to patrol officers and aid them with on-the-job risk-based decision-making. This study also begs the question: What would policing look like if crime maps depicted only cold spots (i.e., lowest-risk areas) instead of the frequently produced hot spot maps? This, too, is worthy of future research.

This study provides data on the types and characteristics of risk information and communication that may elicit either affective or cognitive risk processing. Such data may help police operations attune distinctive types of risk communication to certain aspects of police operations. For example, cognitive risk-based decision-making, which is analytical, conscious, and slow, is thought to potentially lead to more correct and optimum risk-based decisions, compared to affective processing (Evans, 2008). Given the current evidence, types and characteristics of risk information that elicit cognitive risk processing, rather than affective risk processing, may be best for employing well-crafted, developed law enforcement strategies in a geographic location, evaluating the effectiveness of such strategies, or presenting at internal police department operations meetings (Santos, 2014; Santos and Taylor, 2014; Wheeler, 2016). However, risk information that elicits cognitive risk processing may not be best for on-the-job law enforcement risk-based decision-making, as cognitive processing is often a hindrance in situations where rapid decision-making is required (Allen and Thomas, 2011). For example, risk information that elicits cognitive risk processing should probably not be used during calls for service that involve risky situations and suspects, and in which decision-making cannot be controlled or slow. Indeed, affective modes of processing are more important in dynamic risk-based decisions under uncertainty, whereas cognitive evaluations play a larger role in static risky decisions (Figner et al., 2009). Thus, the use of risk information that elicits affective risk processing may be more effectual, depending on the situation and geographic area, for real-time risk-based police decision-making.

Finally, we suggest future inquiries might also want to assess the dual-process processing of risk information communicating crime and public safety conditions, of both police officers and the general public, using neuroscience techniques. Although behavioral research can help to illuminate psychological processes that underlie decision-making and perceptions, it is oftentimes incomplete and does not necessarily provide data on the neurocognitive mechanisms that underlie such processes (Cacioppo, 2002). Indeed, more recent work has begun to utilize neuroscience methods to better and more wholly understand police behavior (e.g., Baldwin et al., 2019; Castro-Toledo et al., 2019; Hashemi et al., 2019). Although there is some evidence on the brain and psychophysiological correlates to dual-process models of processing (see Reyna and Brainerd, 2011), such work has not yet been meaningfully extended to risk information processing. Further, this work also suggests that risk information related to crime and public safety may be processed differently from other categories of risk information when making risk-based decisions, inquiries on risk processing in health, environmental, or other situations may not be relevant or applicable to understanding risk communication and decision-making in the current context. Thus, future work should use neuroscience techniques to better understand the neural mechanisms associated with dual-process risk information processing of crime and public safety data specifically, which may aid in police training, operational training, and better overall risk communication among crime analysts, fusion centers, real-time crime centers, the police department rank-and-file, and their community partners and constituents.

## Data Availability Statement

The raw data supporting the conclusions of this article will be made available by the authors, without undue reservation, to any qualified researcher.

## Ethics Statement

The studies involving human participants were reviewed and approved by Rutgers University Institutional Review Board. The patients/participants provided their electronic informed consent to participate in this study.

## Author Contributions

CB and JC contributed to the design and implementation of the research, to the analysis of the results, and to the writing of the manuscript. Both authors contributed to the article and approved the submitted version.

## Conflict of Interest

The authors declare that the research was conducted in the absence of any commercial or financial relationships that could be construed as a potential conflict of interest.

## References

[B1] Allen A. P. Thomas K. E. (2011). A dual process account of creative thinking. *Creat. Res. J.* 23 109–118. 10.1080/10400419.2011.571183

[B2] Auspurg K. Hinz T. (2014). *Factorial Survey Experiments*, Vol. 175 Thousand Oaks, CA: Sage Publications.

[B3] Baldwin S. Bennell C. Andersen J. P. Semple T. Jenkins B. (2019). Stress-activity mapping: physiological responses during general duty police encounters. *Front. Psychol.* 10:2216. 10.3389/fpsyg.2019.02216 31636582 PMC6788355

[B4] Barger P. Behrend T. S. Sharek D. J. Sinar E. F. (2011). IO and the crowd: frequently asked questions about using mechanical turk for research. *Indus. Organ. Psychol.* 49 11–17.

[B5] Bartneck C. Duenser A. Moltchanova E. Zawieska K. (2015). Comparing the similarity of responses received from studies in Amazon’s Mechanical Turk to studies conducted online and with direct recruitment. *PLoS One* 10:e0121595. 10.1371/journal.pone.0121595 25876027 PMC4397064

[B6] Baughman J. Caplan J. (2020). *Research in Brief: Risk-Based Policing in the Kansas City, Missouri, Police Department.* Available online at: https://www.policechiefmagazine.org/rib-risk-based-policing-kcpd/?ref=ffad9524ee9860aaffe8f7c2e6d65cc7 (accessed June 24, 2020).

[B7] Berryessa C. Lively C. (2019). When a sex offender wins the lottery: social and legal punitiveness toward sex offenders in an instance of perceived injustice. *Psychol. Public Policy Law* 25 181–195. 10.1037/law0000198

[B8] Berryessa C. M. (2017). Jury-eligible public attitudes toward biological risk factors for the development of criminal behavior and implications for capital sentencing. *Crim. Just. Behav.* 44 1073–1100. 10.1177/0093854817716485

[B9] Berryessa C. M. (2018). The effects of psychiatric and “biological” labels on lay sentencing and punishment decisions. *J. Exp. Criminol.* 14 241–256. 10.1007/s11292-018-9322-x

[B10] Berryessa C. M. Krenzer W. L. (2020). The stigma of addiction and effects on community perceptions of procedural justice in drug treatment courts. *J. Drug Issues* 50 303–328. 10.1177/0022042620918950

[B11] Block C. R. Dabdoub M. Fregly S. (1995). *Crime Analysis Through Computer Mapping.* Washington, DC: Police Executive Research Forum.

[B12] Bonkiewicz L. (2015). Bobbies and baseball players: evaluating patrol officer productivity using sabermetrics. *Police Q.* 18 55–78. 10.1177/1098611114560230

[B13] Braga A. A. Turchan B. S. Papachristos A. V. Hureau D. M. (2019). Hot spots policing and crime reduction: an update of an ongoing systematic review and meta-analysis. *J. Exp. Criminol.* 15 289–311. 10.1007/s11292-019-09372-3

[B14] Braga A. A. Weisburd D. (2010). *Policing Problem Places: Crime Hot Spots and Effective Prevention.* Oxford: Oxford University Press.

[B15] Brown S. G. Daus C. S. (2015). The influence of police officers’ decision-making style and anger control on responses to work scenarios. *J. Appl. Res. Memory Cogn.* 4 294–302. 10.1016/j.jarmac.2015.04.001

[B16] Buhrmester M. Kwang T. Gosling S. D. (2011). Amazon’s Mechanical Turk: a new source of inexpensive, yet high-quality, data? *Perspect. Psychol. Sci.* 6 3–5. 10.1177/1745691610393980 26162106

[B17] Cacioppo J. (2002). Social neuroscience: understanding the pieces fosters understanding the whole and vice versa. *Am. Psychol.* 57 19–831. 10.1037/0003-066x.57.11.819 12564179

[B18] Campbell D. E. (2004). Police narrativity in the risk society. *Br. J. Criminol.* 44 695–714. 10.1093/bjc/azh032

[B19] Caplan J. M. (2011). Mapping the spatial influence of crime correlates: a comparison of operationalization schemes and implications for crime analysis and criminal justice practice. *Cityscape* 13 57–83.

[B20] Caplan J. M. Kennedy L. W. (2016). *Risk Terrain Modeling: Crime Prediction and Risk Reduction.* Berkeley, CA: University of California Press.

[B21] Caplan J. M. Kennedy L. W. Miller J. (2011a). Risk terrain modeling: brokering criminological theory and GIS methods for crime forecasting. *Justice Q.* 28 360–381. 10.1080/07418825.2010.486037

[B22] Caplan J. M. Kennedy L. W. Petrossian G. (2011b). Police-monitored CCTV cameras in Newark, NJ: a quasi-experimental test of crime deterrence. *J. Exp. Criminol.* 7 255–274. 10.1007/s11292-011-9125-9

[B23] Caplan J. M. Kennedy L. W. Piza E. L. Barnum J. D. (2020). Using vulnerability and exposure to improve robbery prediction and target area selection. *Appl. Spat. Anal. Policy* 13 1–24.

[B24] Caplan J. M. Moreto W. D. (2012). *GIS Mapping for Public Safety: An Annotated Guide to ArcGIS Version 10 Tools & Procedures.* Newark, NJ: Rutgers Center on Public Security.

[B25] Carson J. V. Wellman A. P. (2018). Problem-oriented policing in suburban low-income housing: a quasi-experiment. *Police Q.* 21 139–170. 10.1177/1098611117744005

[B26] Carter J. G. Carter D. L. Chermak S. McGarrell E. (2017). Law enforcement fusion centers: cultivating an information sharing environment while safeguarding privacy. *J. Police Crim. Psychol.* 32 11–27. 10.1007/s11896-016-9199-4

[B27] Castro-Toledo F. J. Koumaditis K. Mitkidis P. Perea-Garcia J. O. (2019). “Exploring immersive technologies to simulate fear of crime,” in *Proceedings of the 25th ACM Symposium on Virtual Reality Software and Technology*, New York, NY 1–2.

[B28] Chainey S. Tompson L. (2008). *Crime Mapping Case Studies: Practice and Research.* Hoboken, NJ: John Wiley and Sons.

[B29] Chalfin A. McCrary J. (2017). Criminal deterrence: a review of the literature. *J. Econ. Literat.* 55 5–48. 10.1257/jel.20141147

[B30] Coldren J. R. Jr. Huntoon A. Medaris M. (2013). Introducing smart policing: foundations, principles, and practice. *Police Q.* 16 275–286. 10.1177/1098611113497042

[B31] Cope N. (2004). ‘Intelligence led policing or policing led intelligence?’ Integrating volume crime analysis into policing. *Br. J. Criminol.* 44 188–203. 10.1093/bjc/44.2.188

[B32] Dağlar M. Argun U. (2016). Crime mapping and geographical information systems in crime analysis. *J. Hum. Sci.* 13 2208–2221. 10.14687/ijhs.v13i1.3736

[B33] Davis R. C. Ortiz C. W. Euler S. Kuykendall L. (2015). Revisiting “Measuring what matters:” developing a suite of standardized performance measures for policing. *Police Q.* 18 469–495. 10.1177/1098611115598990

[B34] Deane G. Messner S. F. Stucky T. D. McGeever K. Kubrin C. E. (2008). Not ‘islands, entire of themselves’: exploring the spatial context of city-level robbery rates. *J. Quant. Criminol.* 24 363–380. 10.1007/s10940-008-9049-3 19956782 PMC2742481

[B35] Diamond W. D. (1988). The effect of probability and consequence levels on the focus of consumer judgments in risky situations. *J. Consum. Res.* 15 280–283. 10.1086/209165

[B36] Eck J. E. Gersh J. S. Taylor C. (2000). “Finding crime hot spots through repeat address mapping,” in *Analyzing Crime Patterns: Frontiers of Practice*, eds Goldsmith V. McGuire P. G. Mollenkopf J. H. Ross T. A. (Thousand Oaks, CA: SAGE), 49–64. 10.4135/9781452220369.n5

[B37] Ericson R. V. (2007). Rules in policing: five perspectives. *Theoret. Criminol.* 11 367–401. 10.1177/1362480607079583

[B38] Ericson R. V. Haggerty K. D. (1997). *Policing the Risk Society.* Toronto, ON: University of Toronto Press.

[B39] Evans J. S. (2008). Dual-processing accounts of reasoning, judgment, and social cognition. *Annu. Rev. Psychol.* 59 255–278. 10.1146/annurev.psych.59.103006.093629 18154502

[B40] Ferreira M. B. Garcia-Marques L. Sherman S. J. Sherman J. (2006). Automatic and controlled components of judgment and decision making. *J. Pers. Soc. Psychol.* 91 797–813. 17059302 10.1037/0022-3514.91.5.797

[B41] Figner B. Mackinlay R. J. Wilkening F. Weber E. U. (2009). Affective and deliberative processes in risky choice: age differences in risk taking in the Columbia Card Task. *J. Exp. Psychol. Learn. Mem. Cogn.* 35 709–730. 10.1037/a0014983 19379045

[B42] Gabriel U. Greve W. (2003). The psychology of fear of crime. Conceptual and methodological perspectives. *Br. J. Criminol.* 43 600–614. 10.1093/bjc/azg600

[B43] Gaines L. K. Kappeler V. E. Vaughn J. B. (1999). *Policing in America*, 3rd Edn Cincinnati, OH: Anderson Publishing.

[B44] Gibbons F. X. Houlihan A. E. Gerrard M. (2009). Reason and reaction: the utility of a dual-focus, dual-processing perspective on promotion and prevention of adolescent health risk behaviour. *Br. J. Health Psychol.* 14 231–248. 10.1348/135910708x376640 19026095

[B45] Goldsmith V. McGuire P. G. Mollenkopf J. B. Ross T. A. (1999). *Analyzing Crime Patterns: Frontiers of Practice.* Thousand Oaks, CA: Sage Publications.

[B46] Griffin R. J. Dunwoody S. Neuwirth K. (1999). Proposed model of the relationship of risk information seeking and processing to the development of preventive behaviors. *Environ. Res.* 80 S230–S245. 10092438 10.1006/enrs.1998.3940

[B47] Groves R. M. Lyberg L. (2010). Total survey error: past, present, and future. *Public Opin. Q.* 74 849–879. 10.1093/poq/nfq065

[B48] Guilfoyle S. (2016). Getting police performance measurement under control. *Policing* 10 71–87.

[B49] Gutteling J. M. Wiegman O. (2013). *Exploring Risk Communication*, Vol. 8 New York, NY: Springer.

[B50] Harries K. LeBeau J. (2007). Issues in the geographic profiling of crime: review and commentary. *Police Pract. Res.* 8 321–333. 10.1080/15614260701615029

[B51] Harries K. D. (1995). *Mapping Crime: Principle and Practice.* Washington, DC: US Department of Justice, Office of Justice Programs, National Institute of Justice.

[B52] Hashemi M. M. Gladwin T. E. de Valk N. M. Zhang W. Kaldewaij R. van Ast V. (2019). Neural dynamics of shooting decisions and the switch from freeze to fight. *Sci. Rep.* 9 1–10. 30862811 10.1038/s41598-019-40917-8 PMC6414631

[B53] Hendrick L. Vlek C. Oppewal H. (1989). Relative importance of scenario information and frequency information in the judgment of risk. *Acta Psychol.* 72 41–63. 10.1016/0001-6918(89)90050-4

[B54] Hine K. A. Porter L. E. Westera N. J. Alpert G. P. Allen A. (2018). Exploring police use of force decision-making processes and impairments using a Naturalistic decision-making approach. *Crim. Just. Behav.* 45 1782–1801. 10.1177/0093854818789726

[B55] Hodgkinson G. P. Clarke I. (2007). Conceptual note: exploring the cognitive significance of organizational strategizing: a dual-process framework and research agenda. *Hum. Relat.* 60 243–255. 10.1177/0018726707075297

[B56] Kahneman D. (2003). A perspective on judgment and choice: mapping bounded rationality. *Am. Psychol.* 58 697–720. 10.1037/0003-066x.58.9.697 14584987

[B57] Kelly W. R. (2019). *The Future of Crime and Punishment: Smart Policies for Reducing Crime and Saving Money.* Latham, MD: Rowman and Littlefield.

[B58] Kennedy L. W. Caplan J. M. Piza E. L. (2011). Risk clusters, hotspots, and spatial intelligence: risk terrain modeling as an algorithm for police resource allocation strategies. *J. Quantit. Criminol.* 27 339–362. 10.1007/s10940-010-9126-2

[B59] Kennedy L. W. Caplan J. M. Piza E. L. (2018). *Risk-Based Policing: Evidence-Based Crime Prevention with Big Data and Spatial Analytics.* Berkeley, CA: University of California Press.

[B60] Keppel G. Wickens T. D. (2004). *Design and Analysis: A Researcher’s Handbook.* Upper Saddle River, NJ: Pearson Prentice Hall.

[B61] Kruglanski A. W. Orehek E. (2007). Partitioning the domain of social inference: dual mode and systems models and their alternatives. *Annu. Rev. Psychol.* 58 291–316. 10.1146/annurev.psych.58.110405.085629 16968211

[B62] Lerner J. S. Keltner D. (2001). Fear, anger, and risk. *J. Pers. Soc. Psychol.* 81 146–159. 11474720 10.1037//0022-3514.81.1.146

[B63] Loewenstein G. F. Hsee C. K. (2001). Risk as feelings. *Psychol. Bull.* 127 267–286. 11316014 10.1037/0033-2909.127.2.267

[B64] Lundgren R. E. McMakin A. H. (2018). *Risk Communication: A Handbook for Communicating Environmental, Safety, and Health Risks.* Hoboken, NJ: John Wiley and Sons.

[B65] MacInnis B. Krosnick J. A. Ho A. S. Cho M. J. (2018). The accuracy of measurements with probability and nonprobability survey samples: replication and extension. *Public Opin. Q.* 82 707–744. 10.1093/poq/nfy038

[B66] Marx S. M. Weber E. U. Orlove B. S. Leiserowitz A. Krantz D. H. Roncoli C. (2007). Communication and mental processes: experiential and analytic processing of uncertain climate information. *Glob. Environ. Change* 17 47–58. 10.1016/j.gloenvcha.2006.10.004

[B67] McDowall D. Loftin C. (2009). Do US city crime rates follow a national trend? The influence of nationwide conditions on local crime patterns. *J. Quant. Criminol.* 25 307–324. 10.1007/s10940-009-9071-0

[B68] Mesch G. S. (2000). Perceptions of risk, lifestyle activities, and fear of crime. *Deviant Behav.* 21 47–62. 10.1080/016396200266379

[B69] Miller C. (2008). COPLINK CompStat analyzer automates crime data analysis. *Law Enforce. Technol.* 35 136–138.

[B70] Mukherjee K. (2010). A dual system model of preferences under risk. *Psychol. Rev.* 117 243–255. 10.1037/a0017884 20063971

[B71] Nygren T. E. (1998). Reacting to perceived high-and low-risk win–lose opportunities in a risky decision-making task: is it framing or affect or both? *Motivat. Emot.* 22 73–98.

[B72] O’Shea T. Nicholls K. (2003). Police crime analysis: a survey of US police departments with 100 or more sworn personnel. *Police Pract. Res.* 4 233–250. 10.1080/1561426032000113852

[B73] Pasha O. Kroll A. (2016). The effectiveness of CompStat systems: an interrupted time series analysis of crime in US Cities. *Acad. Manag. Proc.* 2016:14713 10.5465/ambpp.2016.14713abstract

[B74] Piza E. L. Caplan J. M. Kennedy L. W. (2017). CCTV as a tool for early police intervention: preliminary lessons from nine case studies. *Security J.* 30 247–265. 10.1057/sj.2014.17

[B75] Pridemore W. A. (2005). A cautionary note on using county-level crime and homicide data. *Homicide Stud.* 9 256–268. 10.1177/1088767905277202

[B76] Rader N. E. (2004). The threat of victimization: a theoretical reconceptualization of fear of crime. *Sociol. Spect.* 24 689–704. 10.1080/02732170490467936

[B77] Randol B. M. (2014). Modelling the influence of organisational structure on crime analysis technology innovations in municipal police departments. *Int. J. Police Sci. Manag.* 16 52–64. 10.1350/ijps.2014.16.1.327

[B78] Reuland M. M. (1997). *Information Management and Crime Analysis.* Washington, DC: Police Executive Research Forum.

[B79] Reyna V. F. Brainerd C. J. (2011). Dual processes in decision making and developmental neuroscience: a fuzzy-trace model. *Dev. Rev.* 31 180–206. 22096268 10.1016/j.dr.2011.07.004 PMC3214669

[B80] Roberts J. (2018). *Public Opinion, Crime, and Criminal Justice.* New York, NY: Routledge.

[B81] Roth R. E. Ross K. S. Finch B. G. Luo W. MacEachren A. M. (2013). Spatiotemporal crime analysis in US law enforcement agencies: current practices and unmet needs. *Govern. Inform. Q.* 30 226–240. 10.1016/j.giq.2013.02.001

[B82] Roth R. E. Ross K. S. MacEachren A. M. (2015). User-centered design for interactive maps: a case study in crime analysis. *ISPRS Int. J. Geo Inform.* 4 262–301. 10.3390/ijgi4010262

[B83] Rouse S. V. (2015). A reliability analysis of Mechanical Turk data. *Comput. Hum. Behav.* 43 304–307. 10.1016/j.chb.2014.11.004

[B84] Santos R. B. (2014). The effectiveness of crime analysis for crime reduction: cure or diagnosis? *J. Contemp. Crim. Just.* 30 147–168. 10.1177/1043986214525080

[B85] Santos R. B. Taylor B. (2014). The integration of crime analysis into police patrol work: results from a national survey of law enforcement agencies. *Policing* 37 501–520. 10.1108/pijpsm-08-2012-0075

[B86] Scott M. S. (2000). *Problem-Oriented Policing: Reflections on the First 20 Years.* Washington, DC: US Department of Justice, Office of Community Oriented Policing Services.

[B87] Slovic P. Peters E. Finucane M. L. Macgregor D. G. (2005). Affect, risk, and decision making. *Health Psychol.* 24 S35–S40. 16045417 10.1037/0278-6133.24.4.S35

[B88] Spiegelhalter D. (2017). Risk and uncertainty communication. *Annu. Rev. Stat. Appl.* 4 31–60.

[B89] Stewart N. Ungemach C. Harris A. J. Bartels D. M. Newell B. R. Paolacci G. (2015). The average laboratory samples a population of 7,300 Amazon Mechanical Turk workers. *Judge. Decis. Making* 10 479–491.

[B90] Sztajnkrycer M. D. Lewinski B. Buhrmaster S. (2010). Leave no one behind: downed-officer rescue and risk perception. *FBI Law Enforce. Bull.* 79 9–14.

[B91] Thompson V. Morsanyi K. (2012). Analytic thinking: do you feel like it? *Mind Soc.* 11 93–105. 10.1007/s11299-012-0100-6

[B92] Van Gelder J. L. de Vries R. E. van der Pligt J. (2009). Evaluating a dual-process model of risk: affect and cognition as determinants of risky choice. *J. Behav. Decis. Mak.* 22 45–61. 10.1002/bdm.610

[B93] Venkatraman V. Payne J. W. Bettman J. R. Luce M. F. Huettel S. A. (2009). Separate neural mechanisms underlie choices and strategic preferences in risky decision making. *Neuron* 62 593–602. 10.1016/j.neuron.2009.04.007 19477159 PMC3213208

[B94] Visschers V. H. M. Wiedemann P. M. Gutscher H. Kurzenhäuser S. Seidl R. Jardine C. G. (2012). Affect-inducing risk communication: current knowledge and future directions. *J. Risk Res.* 15 257–271. 10.1080/13669877.2011.634521

[B95] Vlek C. Stallen P. J. (1980). Rational and personal aspects of risk. *Acta Psychol.* 45 273–300. 10.1016/0001-6918(80)90038-4

[B96] Wang Y. Highhouse S. Lake C. J. Petersen N. L. Rada T. B. (2017). Meta-analytic investigations of the relation between intuition and analysis. *J. Behav. Decis. Mak.* 30 15–25. 10.1002/bdm.1903

[B97] Weisburd D. Groff E. R. Yang S. M. (2012). *The Criminology of Place: Street Segments and Our Understanding of the Crime Problem.* Oxford, UK: Oxford University Press.

[B98] Weisburd D. Mastrofski S. D. McNally A. M. Greenspan R. Willis J. J. (2003). Reforming to preserve: compstat and strategic problem solving in American policing. *Criminol. Public Policy* 2 421–456. 10.1111/j.1745-9133.2003.tb00006.x

[B99] Wheeler A. P. (2016). Tables and graphs for monitoring temporal crime trends: translating theory into practical crime analysis advice. *Int. J. Police Sci. Manag.* 18 159–172. 10.1177/1461355716642781

[B100] Yates J. F. Stone E. R. (1992). “The risk construct,” in *Wiley Series in Human Performance and Cognition*, ed. Yates J. F. (Hoboken, NY: Risk-taking behavior), 1–25.

[B101] You M. Ju Y. (2017). A comprehensive examination of the determinants for food risk perception: focusing on psychometric factors, perceivers’ characteristics, and media use. *Health Commun.* 32 82–91. 10.1080/10410236.2015.1110003 27175517

