# Supplementary material for: Cognitive and Affective Processing of Risk Information: A Survey Experiment on Risk-Based Decision-Making Related to Crime and Public Safety
Source: Front Psychol. 2020 Sep 4;11:2222. doi: 10.3389/fpsyg.2020.02222 (PMC7500154; doi:10.3389/fpsyg.2020.02222)
Supplement: Supplementary file 1 [file Data_Sheet_1.docx]

Supplementary Material

# Supplementary Tables

**Supplementary Table 1.** Main Effects for *Type of Map* on Measures.

| Measure | Pick-Up Point | *F*(1,715) | p-value |
| --- | --- | --- | --- |
| Perceived Risk | North | 1.62 | 0.203 |
|  | South | 2.98 | 0.616 |
|  | East | 2.55 | 0.111 |
|  | West | 0.25 | 0.615 |
| Negative Affect | North | 0.53 | 0.466 |
|  | South | 0.44 | 0.509 |
|  | East | 1.26 | 0.262 |
|  | West | 0.03 | 0.859 |
| Risky Choice | North | 0.00 | 0.967 |
|  | South | 1.14 | 0.285 |
|  | East | 0.03 | 0.867 |
|  | West | 0.09 | 0.764 |
| Ranking | North | 0.18 | 0.668 |
|  | South | 0.30 | 0.581 |
|  | East | 0.00 | 0.981 |
|  | West | 0.40 | 0.525 |

**Supplementary Table 2.** Main Effects for *Type of Risk Data Analysis* on Measures.

| Measure | Pick-Up Point | *F*(8,715) | p-value |
| --- | --- | --- | --- |
| Perceived Risk | North | 1.48 | 0.162 |
|  | South | 1.80 | 0.075 |
|  | East | 1.84 | 0.067 |
|  | West | 1.32 | 0.171 |
| Negative Affect | North | 1.75 | 0.084 |
|  | South | 1.71 | 0.092 |
|  | East | 1.28 | 0.251 |
|  | West | 1.67 | 0.102 |
| Risky Choice | North | 1.35 | 0.216 |
|  | South | 1.31 | 0.240 |
|  | East | 1.40 | 0.194 |
|  | West | 1.18 | 0.309 |
| Ranking | North | 1.63 | 0.113 |
|  | South | 0.73 | 0.392 |
|  | East | 0.02 | 0.877 |
|  | West | 1.18 | 0.309 |

**Supplementary Table 3.** Binary Logistic Regression Results showing the Predictive Power of High *Perceived Risk* and High *Negative Affect* toward the High Likelihood of Being Picked-up at Each Spot (*Risky Choice*) in Control Groups.

| Measure | Pick-Up Point | *Coefficient* | *Odds Ration* | *SE* | *Z* | *p*-value |
| --- | --- | --- | --- | --- | --- | --- |
| High Perceived Risk | High likelihood of being picked up at the North | -1.13 | 3.10 | 0.97 | -1.16 | 0.245 |
|  | High likelihood of being picked up at the South | 0.059 | 1.06 | 1.16 | 0.05 | 0.959 |
|  | High likelihood of being picked up at the East | -1.37 | 3.94 | 0.982 | 1.39 | 0.163 |
|  | High likelihood of being picked up at the West | -1.02 | 2.77 | 0.968 | -1.06 | 0.291 |
| High Negative Affect | High likelihood of being picked up at the North | -1.71 | 5.53 | 1.46 | -1.17 | 0.241 |
|  | High likelihood of being picked up at the South | -0.262 | 1.30 | 1.17 | -0.22 | 0.823 |
|  | High likelihood of being picked up at the East | -1.02 | 2.77 | 0.968 | 1.06 | 0.291 |
|  | High likelihood of being picked up at the West | -1.13 | 3.10 | 0.972 | 1.16 | 0.245 |

**Supplementary Table 4.** Binary Logistic Regression Results showing the Predictive Power of High *Perceived Risk* and High *Negative Affect* toward the High Likelihood of Being Chosen as the Pick-up Spot (*Ranking*) in Control Groups.

| Measure | Pick-Up Point | *Coefficient* | *Odds Ratio* | *SE* | *Z.* | *p*-value |
| --- | --- | --- | --- | --- | --- | --- |
| High Perceived Risk | High likelihood of the North being chosen as the pick-up spot | 1.12 | 3.07 | 1.44 | 0.78 | 0.438 |
|  | High likelihood of the South being chosen as the pick-up spot | 0.600 | 1.82 | 0.948 | 0.63 | 0.527 |
|  | High likelihood of the East being chosen as the pick-up spot | 0.505 | 1.66 | 1.25 | 0.40 | 0.686 |
|  | High likelihood of the West being chosen as the pick-up spot | 0.244 | 1.28 | 1.16 | 0.21 | 0.833 |
| High Negative Affect | High likelihood of the North being chosen as the pick-up spot | -0.499 | 1.65 | 0.838 | -0.59 | 0.552 |
|  | High likelihood of the South being chosen as the pick-up spot | -0.006 | 1.01 | 0.620 | -0.01 | 0.993 |
|  | High likelihood of the East being chosen as the pick-up spot | 1.34 | 3.82 | 0.707 | 1.89 | 0.059 |
|  | High likelihood of the West being chosen as the pick-up spot | 0.055 | 1.06 | 0.731 | 0.08 | 0.940 |

**Supplementary Table 5.** Main and Interaction Effects Comparing Participants’ *Risky Choice* and *Ranking* Ratings Before and After Receiving City-level Risk Information.

| Pick-Up Point | Independent Variable | **Risky Choice** | | **Ranking** | |
| --- | --- | --- | --- | --- | --- |
|  |  | *F* | *p*-value | *F* | *p*-value |
| North | % Risk | 2.54 | 0.111 | 0.06 | 0.810 |
|  | % Risk x Type of Map | 0.23 | 0.631 | 0.00 | 0.980 |
|  | % Risk x Type of Risk Data | 1.27 | 0.254 | 1.08 | 0.374 |
|  | % Risk x Type of Map x Type of Risk Data | 1.46 | 0.159 | 1.19 | 0.300 |
| South | % Risk | 1.09 | 0.296 | 0.03 | 0.852 |
|  | % Risk x Type of Map | 3.47 | 0.063 | 0.64 | 0.423 |
|  | % Risk x Type of Risk Data | 1.09 | 0.366 | 0.62 | 0.780 |
|  | % Risk x Type of Map x Type of Risk Data | 1.08 | 0.372 | 0.40 | 0.934 |
| East | % Risk | 0.17 | 0.678 | 0.06 | 0.808 |
|  | % Risk x Type of Map | 0.03 | 0.870 | 2.73 | 0.100 |
|  | % Risk x Type of Risk Data | 1.19 | 0.296 | 0.90 | 0.525 |
|  | % Risk x Type of Map x Type of Risk Data | 0.81 | 0.605 | 1.47 | 0.155 |
| West | % Risk | 0.40 | 0.526 | 0.26 | 0.610 |
|  | % Risk x Type of Map | 0.01 | 0.934 | 0.83 | 0.364 |
|  | % Risk x Type of Risk Data | 1.81 | 0.063 | 1.06 | 0.388 |
|  | % Risk x Type of Map x Type of Risk Data | 0.76 | 0.651 | 1.53 | 0.133 |

## Supplementary Figures

**
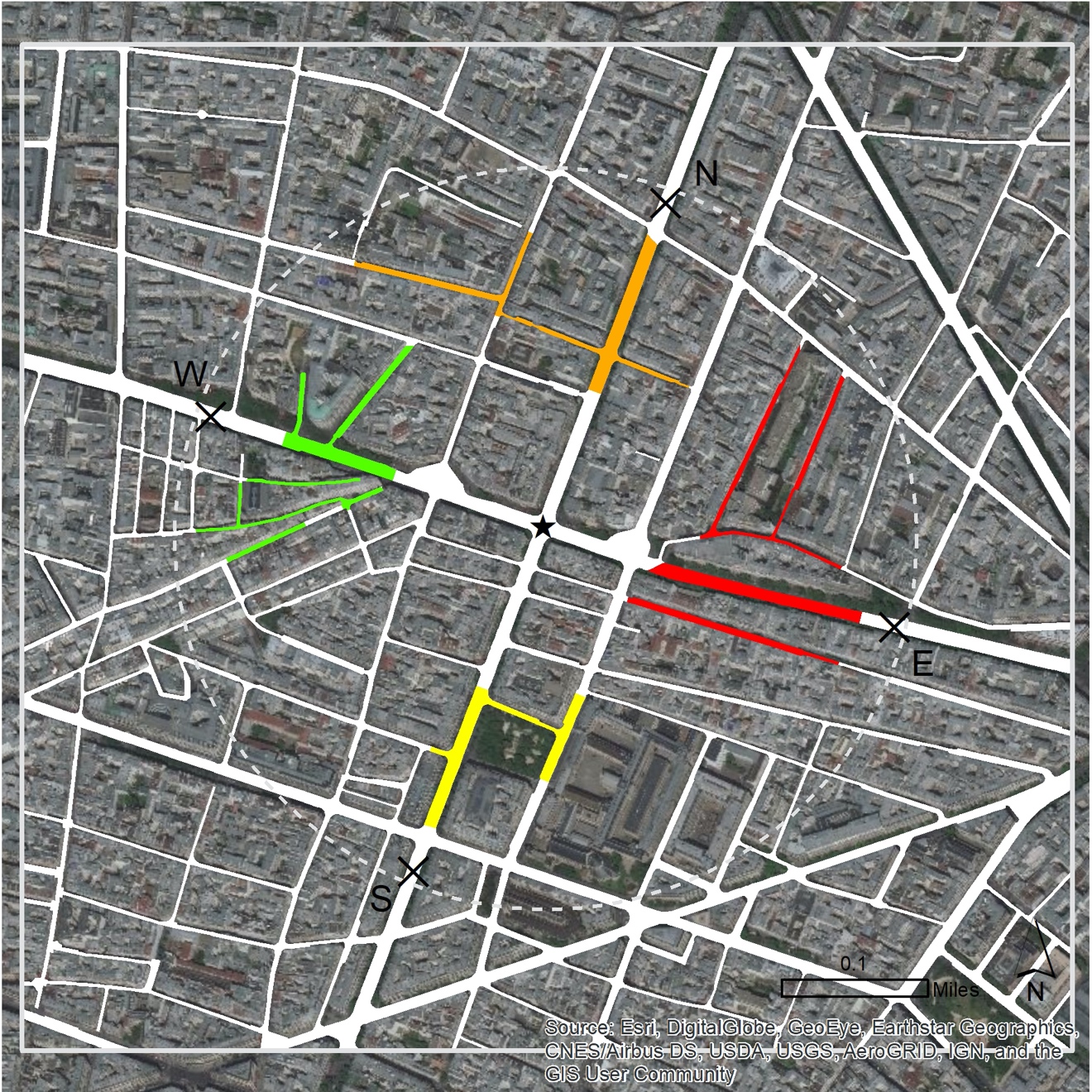
 Supplementary Figure 1.** Risk Information: Thematic Map in Color of Pick-up Area on Satellite Map.

##
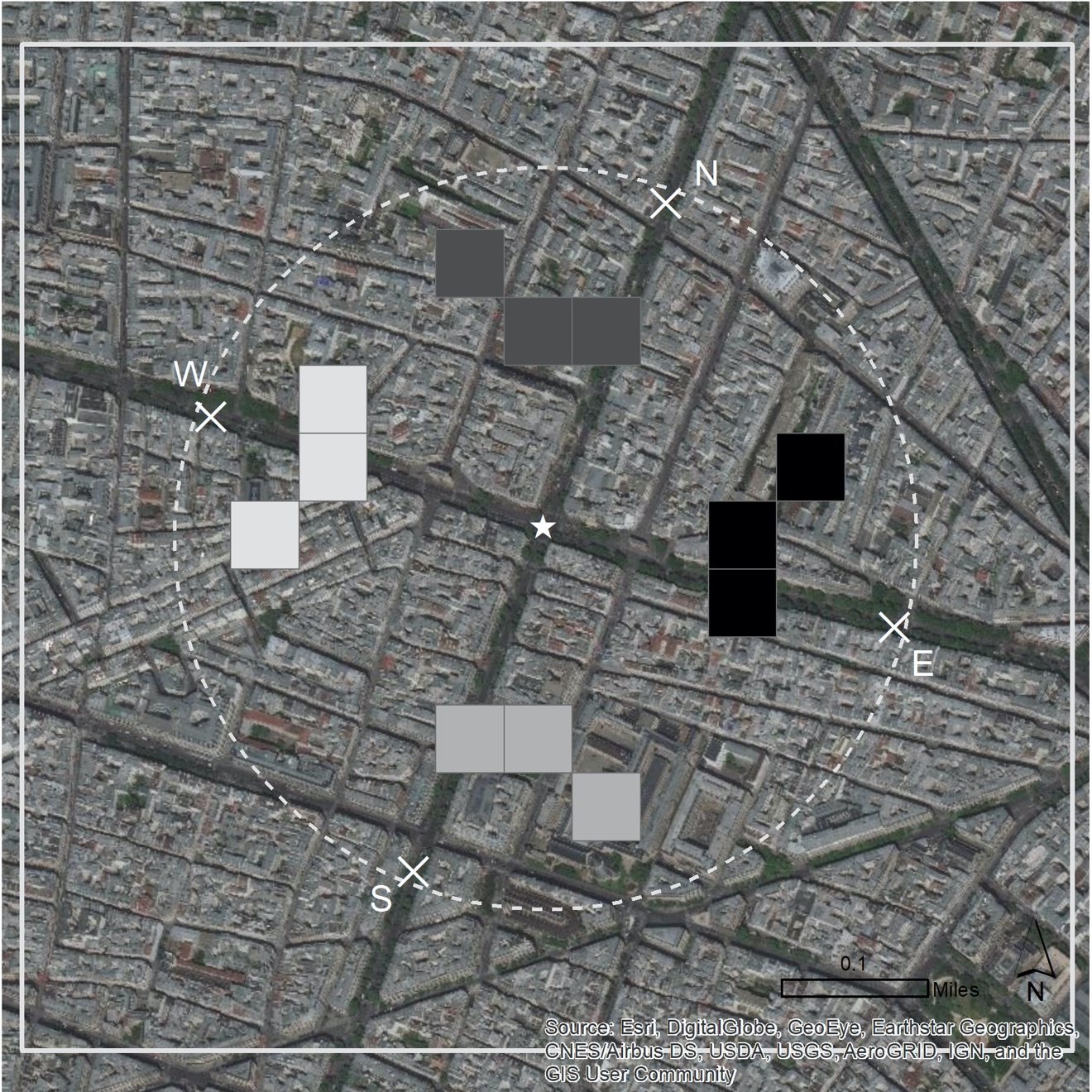


**Supplementary Figure 2.** Risk Information: Hotspot Map in Grayscale of Pick-up Area on Satellite Map.

##
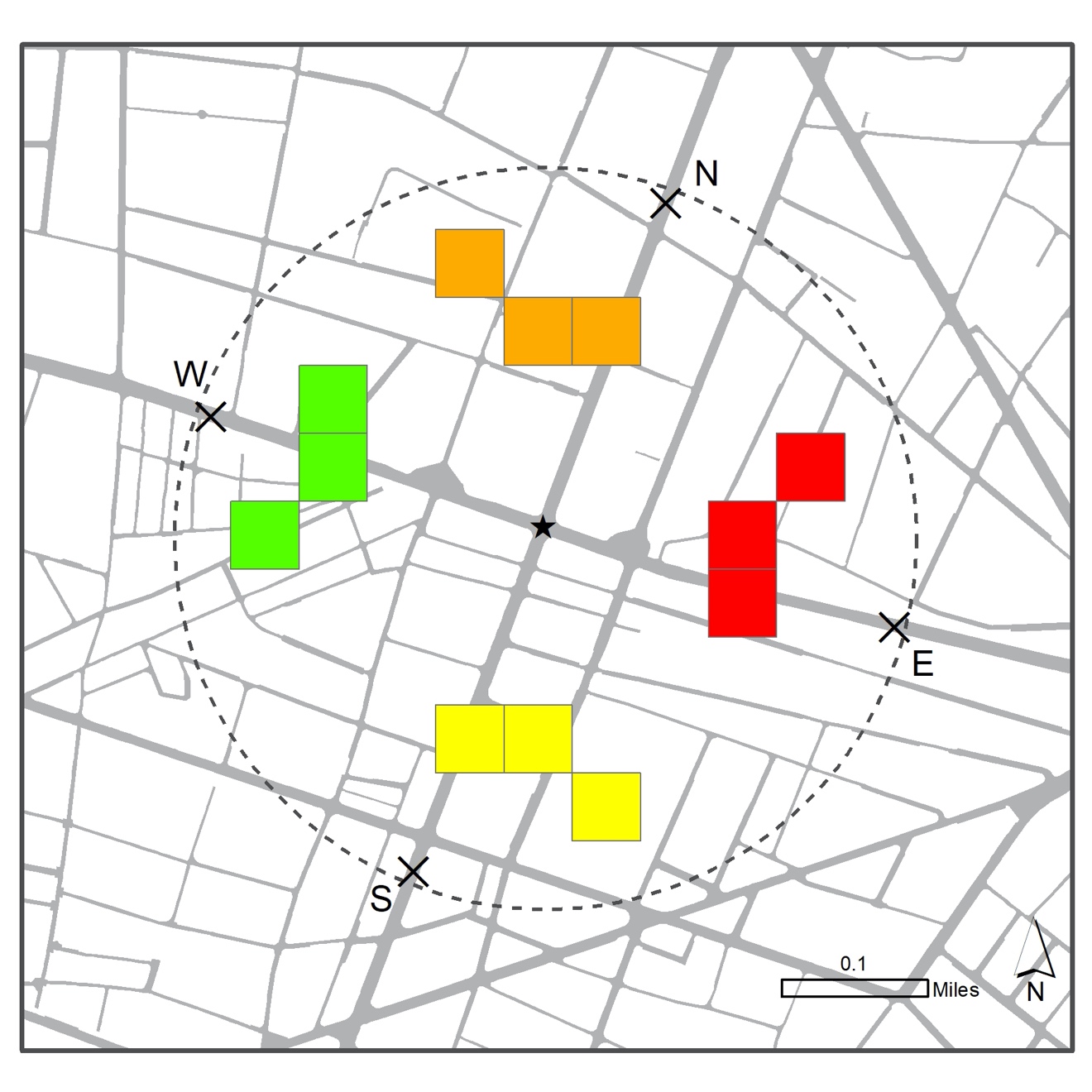


**Supplementary Figure 3.** Risk Information: Hotspot Map in Color of Pick-up Area on Basic Centerline Map.

##
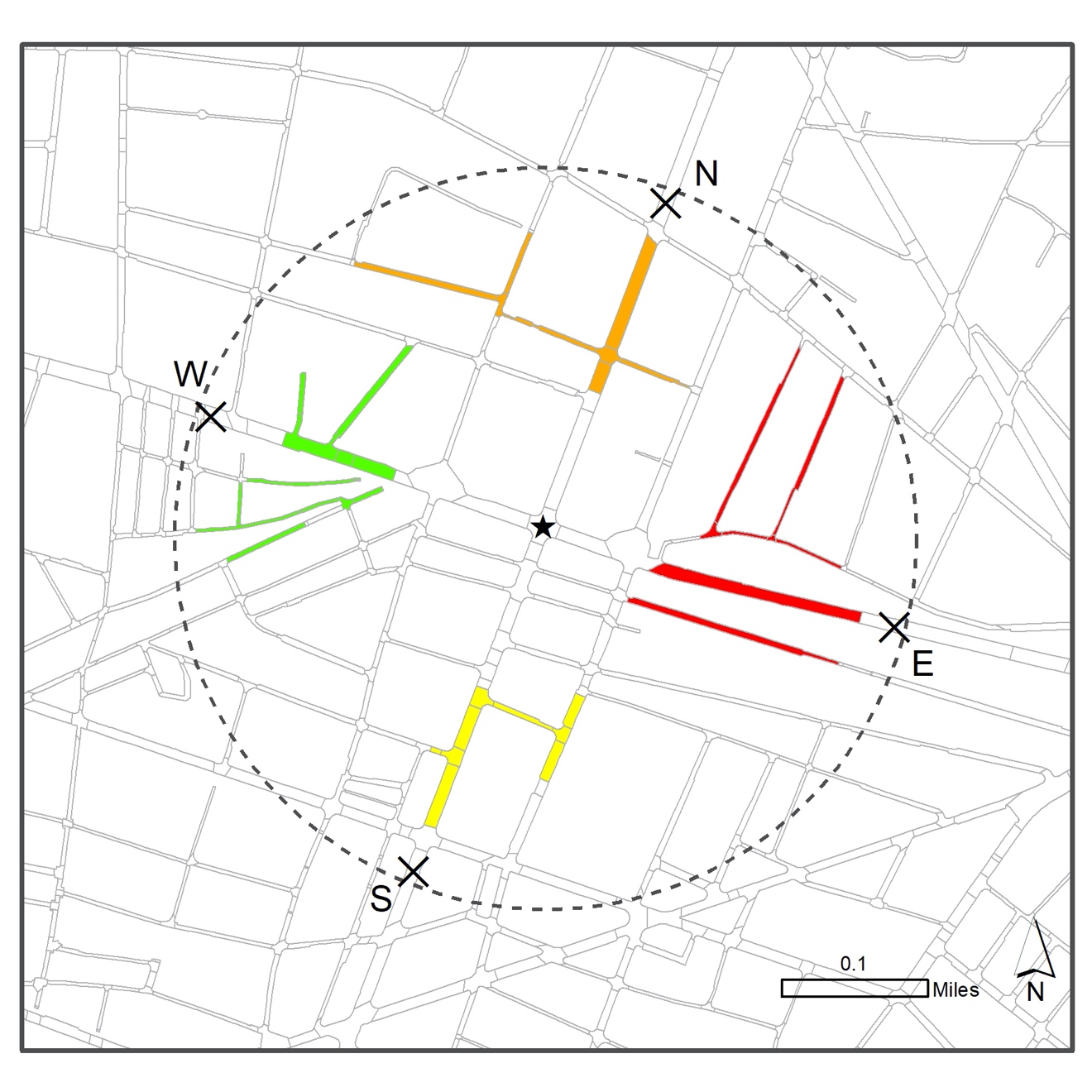


**Supplementary Figure 4.** Risk Information: Thematic Map in Color of Pick-up Area on Basic Centerline Street Map.

##
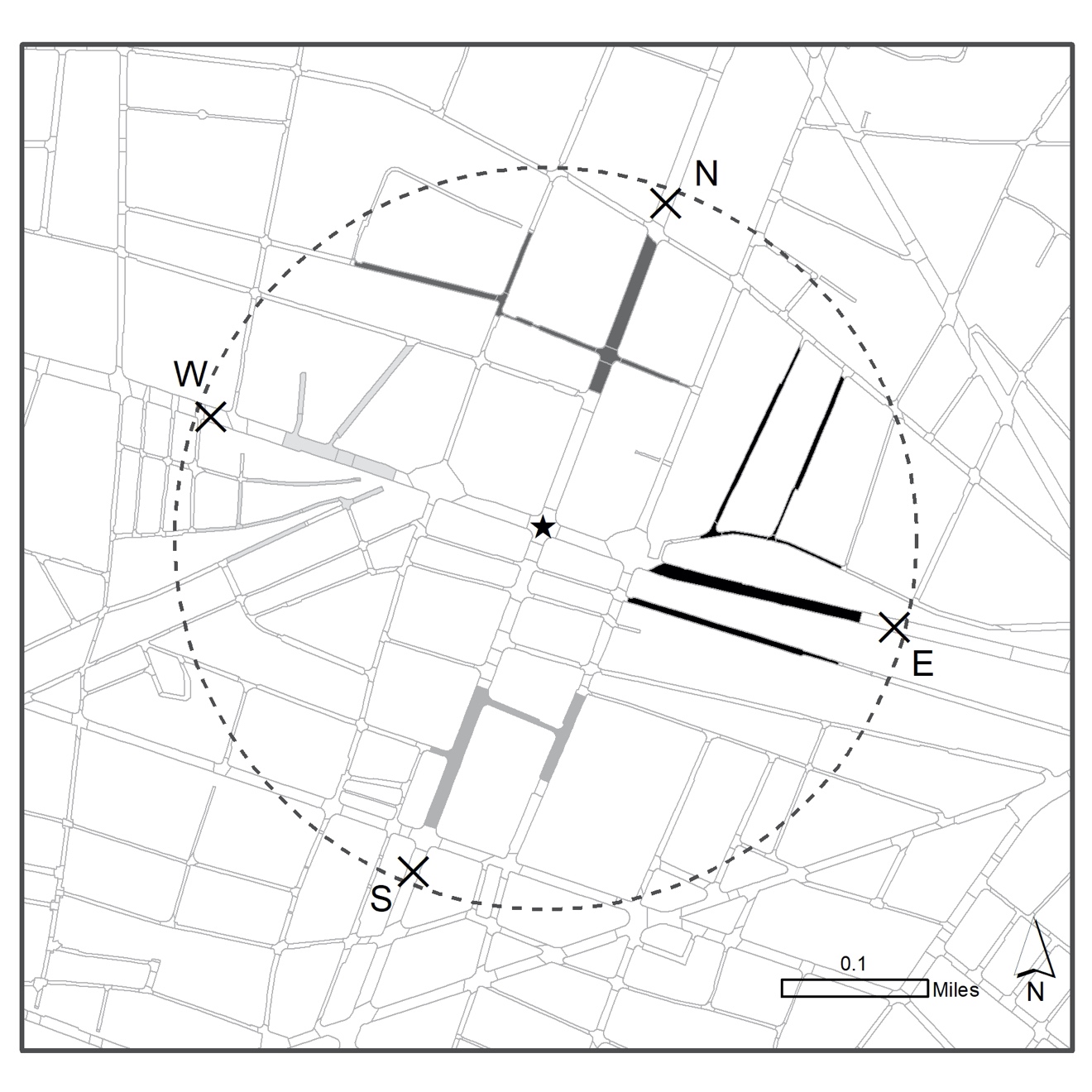


**Supplementary Figure 5.** Risk Information: Thematic Map in Grayscale of Pick-up Area on Basic Centerline Street Map.

**
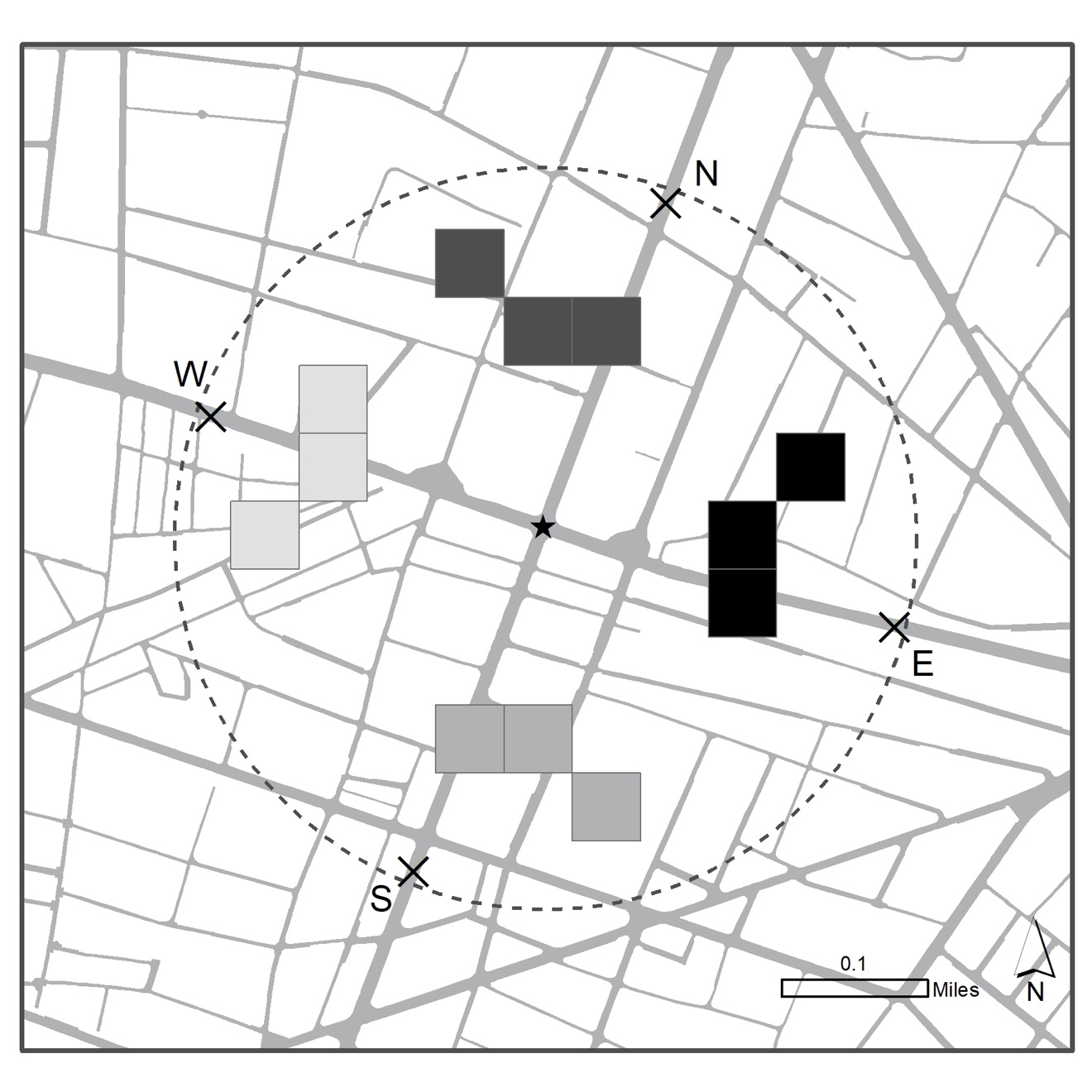
Supplementary Figure 6.** Risk Information: Hotspot Map in Grayscale of Pick-up Area on Basic Centerline Street Map.


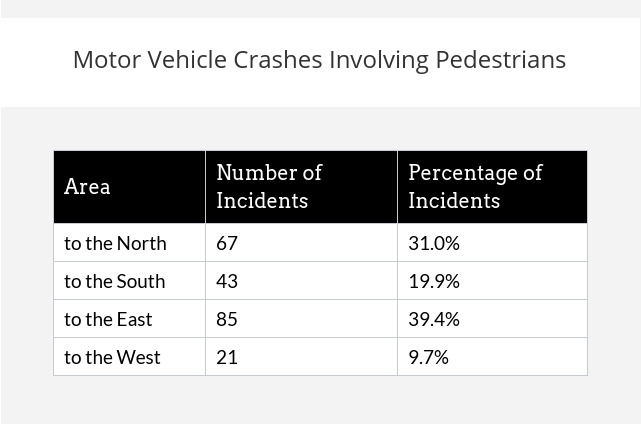


**Supplementary Figure 7.** Risk Information: Table.


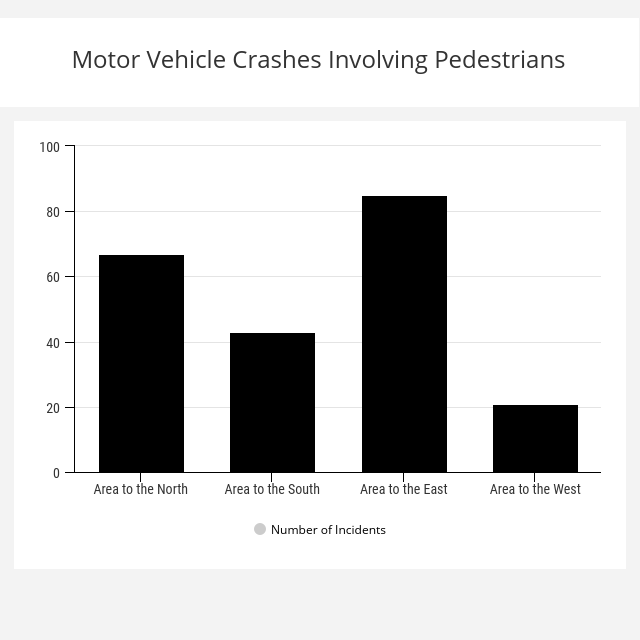


**Supplementary Figure 8.** Risk Information: Bar Graph.

**
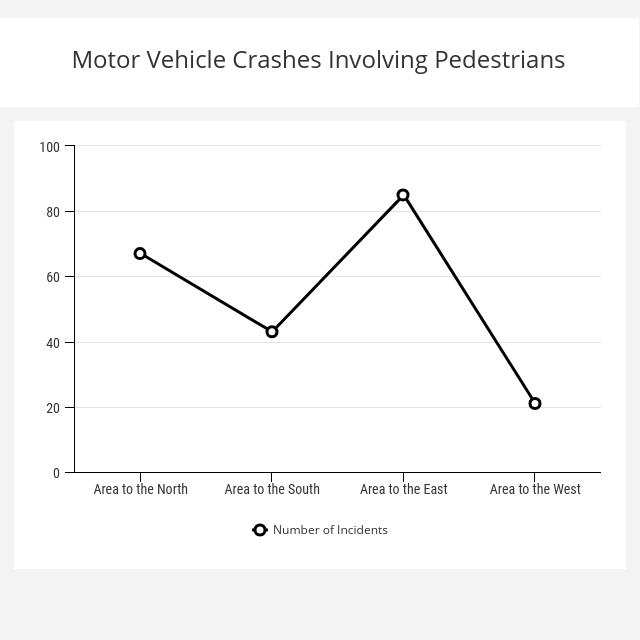
Supplementary Figure 9.** Risk Information: Line Graph.

**
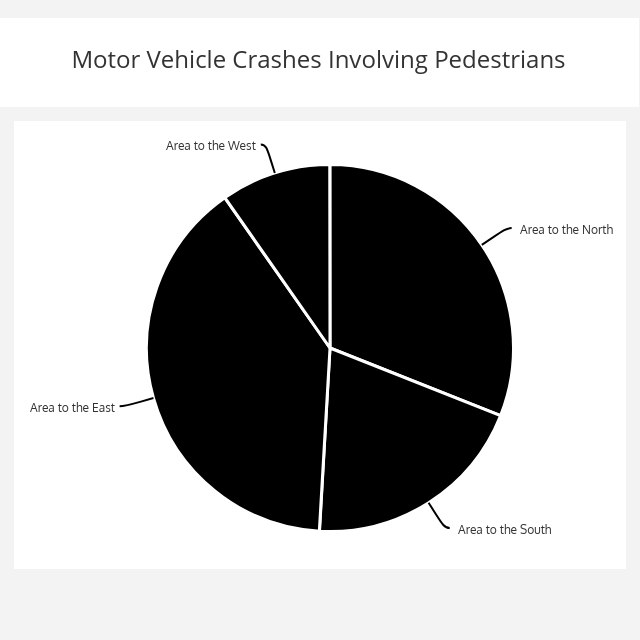
**

**Supplementary Figure 10.** Risk Information: Pie Graph.
